# Multiple wheat genomes reveal global variation in modern breeding

**DOI:** 10.1038/s41586-020-2961-x

**Published:** 2020-11-25

**Authors:** Sean Walkowiak, Liangliang Gao, Cecile Monat, Georg Haberer, Mulualem T. Kassa, Jemima Brinton, Ricardo H. Ramirez-Gonzalez, Markus C. Kolodziej, Emily Delorean, Dinushika Thambugala, Valentyna Klymiuk, Brook Byrns, Heidrun Gundlach, Venkat Bandi, Jorge Nunez Siri, Kirby Nilsen, Catharine Aquino, Axel Himmelbach, Dario Copetti, Tomohiro Ban, Luca Venturini, Michael Bevan, Bernardo Clavijo, Dal-Hoe Koo, Jennifer Ens, Krystalee Wiebe, Amidou N’Diaye, Allen K. Fritz, Carl Gutwin, Anne Fiebig, Christine Fosker, Bin Xiao Fu, Gonzalo Garcia Accinelli, Keith A. Gardner, Nick Fradgley, Juan Gutierrez-Gonzalez, Gwyneth Halstead-Nussloch, Masaomi Hatakeyama, Chu Shin Koh, Jasline Deek, Alejandro C. Costamagna, Pierre Fobert, Darren Heavens, Hiroyuki Kanamori, Kanako Kawaura, Fuminori Kobayashi, Ksenia Krasileva, Tony Kuo, Neil McKenzie, Kazuki Murata, Yusuke Nabeka, Timothy Paape, Sudharsan Padmarasu, Lawrence Percival-Alwyn, Sateesh Kagale, Uwe Scholz, Jun Sese, Philomin Juliana, Ravi Singh, Rie Shimizu-Inatsugi, David Swarbreck, James Cockram, Hikmet Budak, Toshiaki Tameshige, Tsuyoshi Tanaka, Hiroyuki Tsuji, Jonathan Wright, Jianzhong Wu, Burkhard Steuernagel, Ian Small, Sylvie Cloutier, Gabriel Keeble-Gagnère, Gary Muehlbauer, Josquin Tibbets, Shuhei Nasuda, Joanna Melonek, Pierre J. Hucl, Andrew G. Sharpe, Matthew Clark, Erik Legg, Arvind Bharti, Peter Langridge, Anthony Hall, Cristobal Uauy, Martin Mascher, Simon G. Krattinger, Hirokazu Handa, Kentaro K. Shimizu, Assaf Distelfeld, Ken Chalmers, Beat Keller, Klaus F. X. Mayer, Jesse Poland, Nils Stein, Curt A. McCartney, Manuel Spannagl, Thomas Wicker, Curtis J. Pozniak

**Affiliations:** 1 Crop Development Centre, University of Saskatchewan, Saskatoon, Saskatchewan Canada; 2 Grain Research Laboratory, Canadian Grain Commission, Winnipeg, Manitoba Canada; 3 Department of Plant Pathology, Kansas State University, Manhattan, KS USA; 4 Leibniz Institute of Plant Genetics and Crop Plant Research (IPK) Gatersleben, Seeland, Germany; 5 Helmholtz Zentrum München—German Research Center for Environmental Health, Neuherberg, Germany; 6 Aquatic and Crop Resource Development, National Research Council Canada, Saskatoon, Saskatchewan Canada; 7 John Innes Centre, Norwich Research Park, Norwich, UK; 8 Department of Plant and Microbial Biology, University of Zurich, Zurich, Switzerland; 9 Morden Research and Development Centre, Agriculture and Agri-Food Canada, Morden, Manitoba Canada; 10 Department of Computer Science, University of Saskatchewan, Saskatoon, Saskatchewan Canada; 11 Brandon Research and Development Centre, Agriculture and Agri-Food Canada, Brandon, Manitoba Canada; 12 Genomics/Transcriptomics group, Functional Genomics Center Zurich, Zurich, Switzerland; 13 Department of Evolutionary Biology and Environmental Studies, University of Zurich, Zurich, Switzerland; 14 Institute of Agricultural Sciences, ETHZ, Zurich, Switzerland; 15 Kihara Institute for Biological Research, Yokohama City University, Yokohama, Japan; 16 Life Sciences Department, Natural History Museum, London, UK; 17 Earlham Institute, Norwich Research Park, Norwich, UK; 18 The John Bingham Laboratory, NIAB, Cambridge, UK; 19 Department of Agronomy and Plant Genetics, University of Minnesota, Saint Paul, MN USA; 20 Global Institute for Food Security, University of Saskatchewan, Saskatoon, Saskatchewan Canada; 21 School of Plant Sciences and Food Security, Tel Aviv University, Ramat Aviv, Israel; 22 Department of Entomology, University of Manitoba, Winnipeg, Manitoba Canada; 23 Institute of Crop Science, NARO, Tsukuba, Japan; 24 Centre for Biodiversity Genomics, University of Guelph, Guelph, Ontario Canada; 25 National Institute of Advanced Industrial Science and Technology (AIST), Tokyo, Japan; 26 Laboratory of Plant Genetics, Graduate School of Agriculture, Kyoto University, Kyoto, Japan; 27 Humanome Lab, Tokyo, Japan; 28 Global Wheat Program, International Maize and Wheat Improvement Center (CIMMYT), Texcoco, Mexico; 29 Montana BioAg, Missoula, MT USA; 30 Australian Research Council Centre of Excellence in Plant Energy Biology, School of Molecular Sciences, University of Western Australia, Perth, Western Australia Australia; 31 Ottawa Research and Development Centre, Agriculture and Agri-Food Canada, Ottawa, Ontario Canada; 32 Agriculture Victoria, AgriBio, Centre for AgriBioscience, Bundoora, Victoria Australia; 33 Syngenta, Durham, NC USA; 34 School of Agriculture, Food and Wine, University of Adelaide, Adelaide, South Australia Australia; 35 German Centre for Integrative Biodiversity Research (iDiv) Halle-Jena-Leipzig, Leipzig, Germany; 36 Biological and Environmental Science & Engineering Division, King Abdullah University of Science and Technology, Thuwal, Saudi Arabia; 37 Graduate School of Life and Environmental Sciences, Kyoto Prefectural University, Kyoto, Japan; 38 Institute of Evolution and Department of Evolutionary and Environmental Biology, University of Haifa, Haifa, Israel; 39 School of Life Sciences Weihenstephan, Technical University of Munich, Freising, Germany; 40 Center for Integrated Breeding Research (CiBreed), Georg-August-University Göttingen, Göttingen, Germany

**Keywords:** Structural variation, Comparative genomics, Haplotypes, Plant breeding

## Abstract

Advances in genomics have expedited the improvement of several agriculturally important crops but similar efforts in wheat (*Triticum* spp.) have been more challenging. This is largely owing to the size and complexity of the wheat genome^1^, and the lack of genome-assembly data for multiple wheat lines^2,3^. Here we generated ten chromosome pseudomolecule and five scaffold assemblies of hexaploid wheat to explore the genomic diversity among wheat lines from global breeding programs. Comparative analysis revealed extensive structural rearrangements, introgressions from wild relatives and differences in gene content resulting from complex breeding histories aimed at improving adaptation to diverse environments, grain yield and quality, and resistance to stresses^4,5^. We provide examples outlining the utility of these genomes, including a detailed multi-genome-derived nucleotide-binding leucine-rich repeat protein repertoire involved in disease resistance and the characterization of *Sm1*^6^, a gene associated with insect resistance. These genome assemblies will provide a basis for functional gene discovery and breeding to deliver the next generation of modern wheat cultivars.

## Abstract (web summary)

Comparison of multiple genome assemblies from wheat reveals extensive diversity that results from the complex breeding history of wheat and provides a basis for further potential improvements to this important food crop.

## Main

Wheat is a staple food across all parts of the world and is one of the most widely grown and consumed crops^7^. As the human population continues to grow, wheat production must increase by more than 50% over current levels by 2050 to meet demand^7^. Efforts to increase wheat production may be aided by comprehensive genomic resources from global breeding programs to identify within-species allelic diversity and determine the best allele combinations to produce superior cultivars^2,8^.

Two species dominate current global wheat production: allotetraploid (AABB) durum wheat (*Triticum turgidum* ssp. *durum*), which is used to make couscous and pasta^9^, and allohexaploid (AABBDD) bread wheat (*Triticum aestivum*), used for making bread and noodles. A, B and D in these designations correspond to separate subgenomes derived from three ancestral diploid species with similar but distinct genome structure and gene content that diverged between 2.5 and 6 million years ago^10^. The large genome size (16 Gb for bread wheat), high sequence similarity between subgenomes and abundance of repetitive elements (about 85% of the genome) hampered early wheat genome-assembly efforts^3^. However, chromosome-level assemblies have recently become available for both tetraploid^11,12^ and hexaploid wheat^1,13^. Although these genome assemblies are valuable resources, they do not fully capture within-species genomic variation that can be used for crop improvement, and comparative genome data from multiple individuals is still needed to expedite bread wheat research and breeding. Until now, comparative genomics of multiple bread wheat lines have been limited to exome-capture sequencing^4,5,14^, low-coverage sequencing^2^ and whole-genome scaffolded assemblies^13,15–17^. Here we report multiple reference-quality genome assemblies and explore genome variation that, owing to past breeder selection, differs greatly between bread wheat lines. These genome assemblies usher a new era for bread wheat and equip researchers and breeders with the tools needed to improve bread wheat and meet future food demands.

## Global variation in wheat genomes

**Fig. 1:**
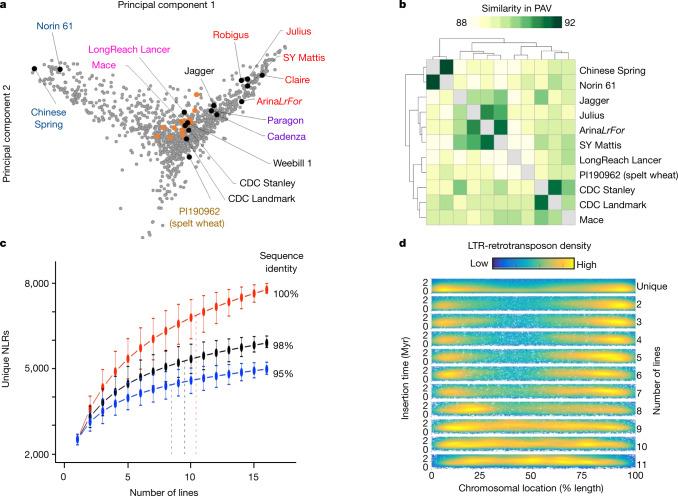
Patterns of variation in the wheat genome. **a**, Principal component analysis of polymorphisms from exome-capture sequencing of about 1,200 lines (grey markers), 16 lines from whole-genome shotgun resequencing (orange markers) and our new assemblies (black markers). Text colours reflect different geographical locations and winter or spring growth. **b**, Dendrogram of pairwise Jaccard similarities for gene PAV between all RQA assemblies. **c**, Number of unique NLRs at different per cent identity cut-offs as the number of genomes increases. Dashed vertical lines represent 90% of the NLR complement. Markers indicate the mean values of all permutations of the order of adding genomes. Whiskers show maximum and minimum values based on one million random permutations. **d**, Chromosomal location versus insertion age distribution of unique to (reading downward) increasingly shared syntenic full-length LTR retrotransposons.

**Extended Data Fig. 1:**
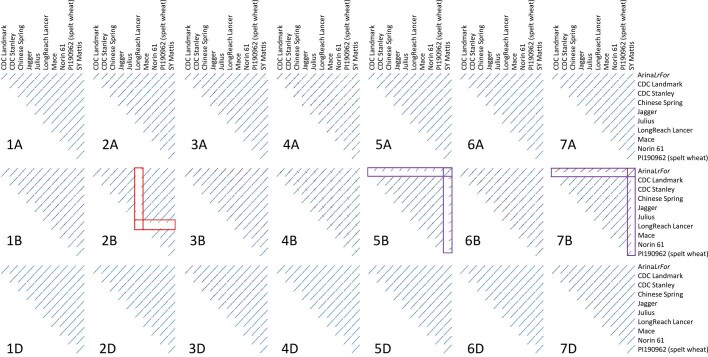
Chromosome-scale collinearity between the RQA. Genomes were aligned chromosome by chromosome using MUMmer and are represented as dot plots. The introgression on chromosome 2B of LongReach Lancer (red rectangles) and 5B/7B translocation in SY Mattis and Arina*LrFor* (purple rectangles) are indicated.

**Extended Data Fig. 2:**
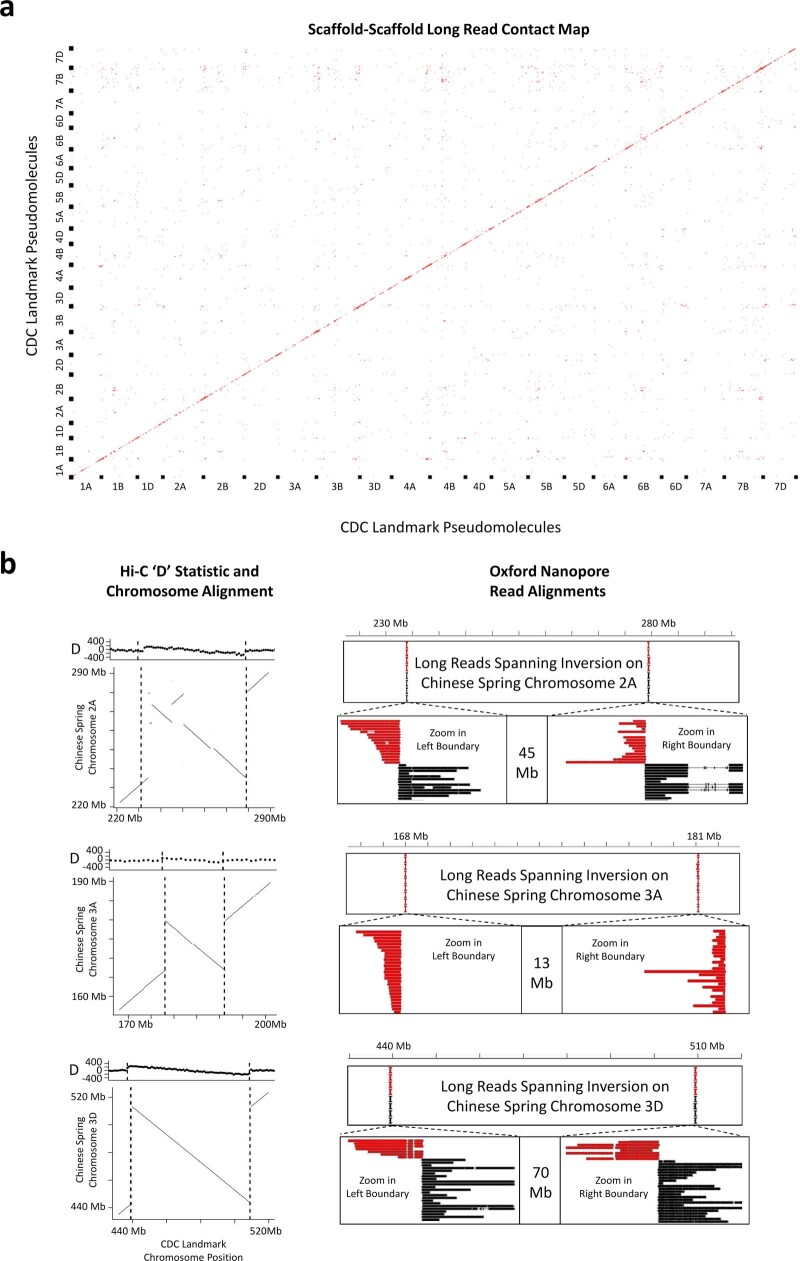
Evaluation of the CDC Landmark RQA using Oxford Nanopore Long Reads. **a**, Scaffold-scaffold long read contact map showing shared read IDs between scaffold ends along the ordered scaffolds in the CDC Landmark pseudomolecules. The diagonal pattern indicates that adjacent scaffolds share the same long reads and are therefore properly ordered and oriented by Hi-C in the RQA. **b**, Characterization of inversion events on chromosomes 2A, 3A, and 3D. The directionality biases estimated from alignments of Hi-C data against Chinese Spring (left, top), and chromosome alignment of the inversion events between CDC Landmark and Chinese Spring RQAs (left, bottom) are shown. Long reads spanning the inversion events and magnified views of the reads aligning to the left and right boundaries of the inversions (right) are provided.

To expand on the genome assembly of wheat for Chinese Spring^1^, we generated ten reference-quality pseudomolecule assemblies (RQAs) and five scaffold-level assemblies of hexaploid wheat (Supplementary Note 1, Supplementary Tables 1–3). For each RQA, we performed de novo assembly of contigs (contig N50 > 48 kb) that were combined into scaffolds (N50 > 10 Mb) spanning more than 14.2 Gb (Supplementary Note 1). The completeness of the genomes was supported by a universal single-copy orthologue (BUSCO) analysis that identified more than 97% of the expected gene content in each genome (Supplementary Note 1). More than 94% of the scaffolds were ordered, oriented and curated using 10X Genomics linked reads and three-dimensional chromosome conformation capture sequencing (Hi-C) to generate 21 pseudomolecules, as done previously for wheat^1,12^ and barley (*Hordeum vulgare*)^18^. The size and structure of the genomes were similar to that of Chinese Spring, and we observed high collinearity between the pseudomolecules (Extended Data Fig. 1). We also independently validated the scaffold placement and orientation in the pseudomolecule assembly of CDC Landmark by Oxford Nanopore long-read sequencing (Extended Data Fig. 2a, Supplementary Note 2). To complement the RQAs, we generated scaffold-level assemblies of five additional bread wheat lines (Supplementary Note 1). To determine the global context of the 15 assemblies, we combined our data with existing datasets^4,5,19^ (Fig. 1a, Supplementary Table 4). The genetic relationships were in agreement with those reported in previous studies^4,5^ and reflected pedigree, geographical location and growth habit (that is, spring versus winter type). There was also a clear separation between the newly assembled genomes and Chinese Spring, supporting that they capture geographical and historical variation not represented in the Chinese Spring assembly.

## Polyploidy and CNV drive gene diversification

**Extended Data Fig. 3:**
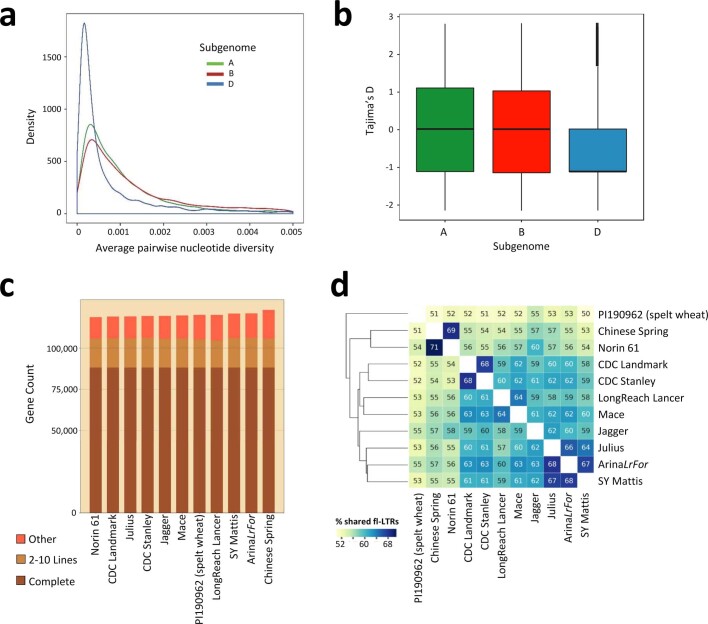
Diversity of genes and TEs. **a**, Average pairwise genetic diversity of the homeologues (coding sequences only) of the A, B and D subgenomes. The mode of the A, B and D subgenome is 0.00057, 0.00082, and 0.0002, respectively. **b**, Tajima’s D estimates of coding sequences for each wheat subgenome. The lower and upper range of the boxplot hinges correspond to the first and third quartiles (the 25th and 75th percentiles). Boxplots show centre line, median; box limits, upper and lower quartiles; whiskers, 1.5 × interquartile range. **c**, Total gene counts and orthologues for the RQA. Genes in orthologous groups with exactly one gene for each line (Complete; dark brown), genes contained in unambiguous orthologous groups missing an orthologue for at least one line, that is, PAV (2-10 Lines; light brown), and genes with ambiguous orthologues or CNV (Other; pink) are indicated. **d**, Per cent of pairwise shared syntenic fl-LTRs between wheat lines.

Single-nucleotide polymorphisms (SNPs), insertions or deletions (indels), presence/absence variation (PAV) and gene copy number variation (CNV) influence agronomically important traits. This is particularly true for polyploid species such as wheat, in which gene redundancy can buffer the effect of genome variation^17^. To assess gene content, we projected around 107,000 high-confidence gene models from Chinese Spring^1^ onto the RQAs (Supplementary Note 3). The total number of projected genes exhibited a narrow range, between 118,734 and 120,967 (Supplementary Table 5). We identified orthologous groups among projected genes and used the alignment of the orthologous groups to examine SNPs in coding sequences (Supplementary Note 3). The peak positions of nucleotide diversity across the three subgenomes were highly similar to those reported in previous studies^20^, supporting a strong representation of breeding diversity within the RQAs (Extended Data Fig. 3a, b). The correlation of synonymous nucleotide diversity *π* (*r* = 0.11–0.29) and Tajima’s *D* (*r* = 0.02–0.06) between homeologues was low (Supplementary Tables 6–8). This suggested that polyploidization increased the number of targets of selection and contributed to broad adaptation of bread wheat, as in wild polyploid plant species^20–22^. Further investigation of orthologous groups indicated that 88.1% were unambiguous (clusters containing at most one member in each cultivar) (Extended Data Fig. 3c, Supplementary Table 5). Orthologous groups comprising exactly one gene in each line (‘complete’) were the most frequent (approximately 73.5% of genes per cultivar), suggesting strong retention of orthologous genes within the ten RQAs. The residual genes represented either singleton genes with no reciprocal best BLAST hits or genes located in complex clusters in at least one cultivar. Roughly 12% of genes showed PAVs, and their clustering resulted in relationships (Fig. 1b) that were consistent with SNP-based phylogenetic similarities (Fig. 1a). In addition, approximately 26% of the projected genes were found in tandem duplications, indicating that CNV is a strong contributor of genetic variation in wheat.

**Extended Data Fig. 4:**
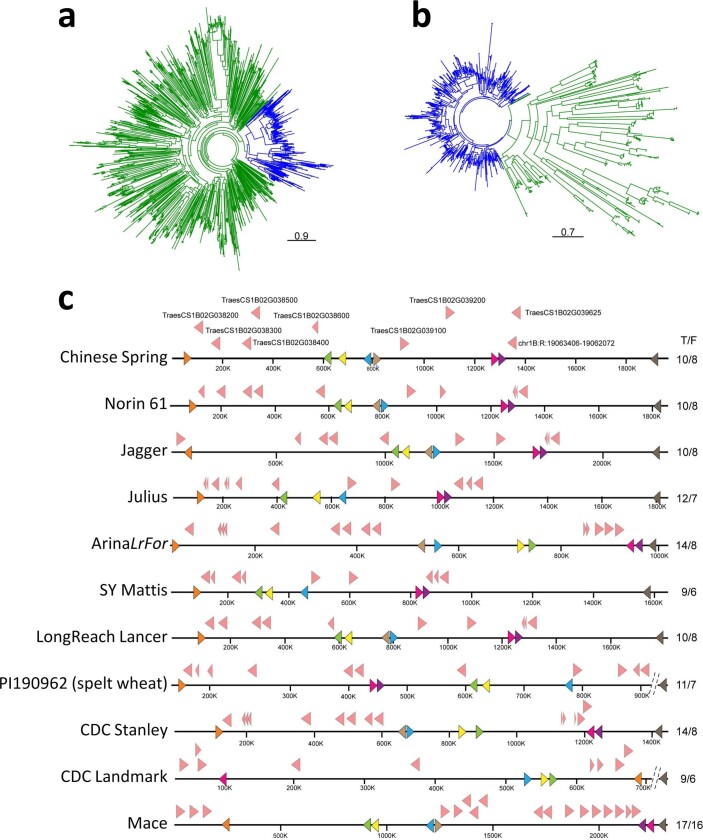
Evolutionary relationships among PPR and mTERF gene sequences. **a**, The *RFL* clade is in blue and all remaining P-class PPRs are in green. **b**, Clustered mTERF sequences are in blue and the remaining mTERFs are shown in green. The scale bar represents number of substitutions per site. **c**, Sequence inversions and copy number variation at the *Rf3* locus on chromosome 1B. *RFL* genes are shown as light pink triangles above the chromosome scale. Conserved non-PPR genes used as syntenic anchors are shown on the chromosome scale as coloured triangles. The total number (T) and the number of putatively functional *RFL* genes with 10 or more PPR motifs (F) are indicated on the right side of each panel.

To provide an example of gene expansion on emerging breeding targets, we performed a more detailed analysis of the restorer of fertility (*Rf*) gene families (Supplementary Note 4). *Rf* genes are involved in restoring pollen fertility in hybrid breeding programs^23^, and we identified a previously undescribed clade within the mitochondrial transcription termination factor (mTERF) family (Supplementary Table 9), which has recently been implicated in fertility restoration in barley^24^. Of note, this clade shows evolutionary patterns similar to those of *Rf-*like pentatricopeptide repeat (PPR) proteins, representatives of which are associated with *Rf3*, a major locus used in hybrid wheat breeding programs (Extended Data Fig. 4). Although wheat is currently not a hybrid crop, there is substantial interest in *Rf* genes and their potential application in hybrid wheat production systems^25^. To our knowledge, no *Rf* genes have been cloned in wheat and our analysis of *Rf* genes in multiple RQAs and identification of an *Rf* clade in wheat is an important step forward in tackling the challenges of hybrid wheat breeding.

## The wheat NLR repertoire

To further exemplify the use of multi-genome comparisons for characterizing agronomically relevant gene families, we examined gene expansion in nucleotide-binding leucine-rich repeat (NLR) proteins, which are major components of the innate immune system and are often causal genes for disease resistance in plants^26,27^. We performed de novo annotation of loci that contain conserved NLR motifs (NB-ARC–leucine-rich repeat) and identified around 2,500 loci with NLR signatures in each RQA (Supplementary Tables 10, 11). A redundancy analysis showed that only 31–34% of the NLR signatures are shared across all genomes, and the number of unique signatures ranged from 22 to 192 per wheat cultivar. We estimated the number of unique NLR signatures that can be detected by incrementally adding more wheat genomes to the dataset; this revealed that 90% of the NLR complement is reached at between 8 (considering 95% sequence identity) and 11 wheat lines (considering 100% protein sequence identity) (Fig. 1c). The total NLR complement of all wheat lines consisted of 5,905 (98% identity) to 7,780 (100% identity) unique NLR signatures, highlighting the size and complexity of the repertoire of receptors involved in disease resistance.

## Transposon signatures identify introgressions

**Fig. 2:**
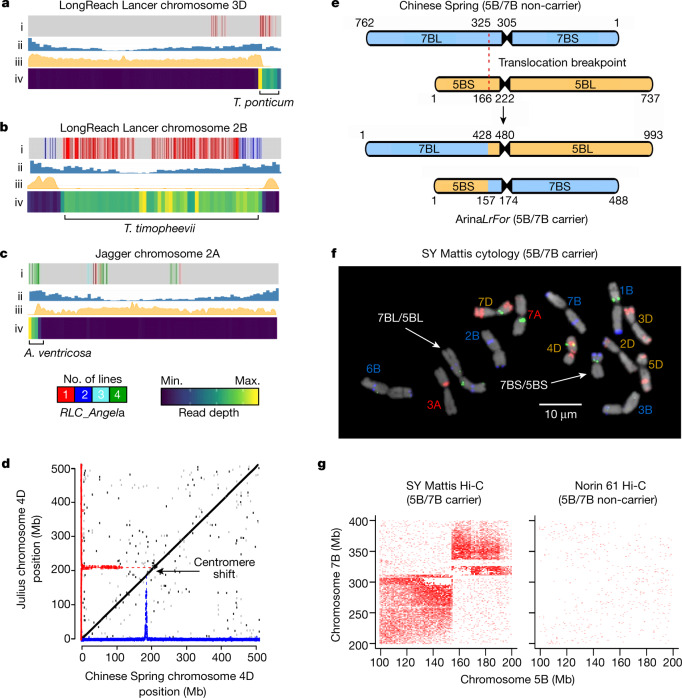
Introgressions and large-scale structural variation in wheat. **a**–**c**, *T. ponticum* introgression on chromosome 3D in LongReach Lancer (**a**), *T. timopheevi* introgression on chromosome 2B in LongReach Lancer (**b**) and *A. ventricosa* introgression on chromosome 3D in Jagger (**c**). Track i, map of polymorphic RLC-*Angela* retrotransposon insertions (legend at bottom); track ii, density of projected gene annotations from Chinese Spring (blue bars, scaled to maximum value); track iii, per cent identity to Chinese Spring based on chromosome alignment (yellow; scale is 0–100%); track iv, read depth of wheat wild relatives (blue–yellow heat map; legend at bottom). **d**, Dot plot alignment showing chromosome-level collinearity (black) with relative density of CENH3 ChIP–seq mapped to 100-kb bins for Chinese Spring (blue) and Julius (red); the arrow indicates a centromere shift. **e**, Robertsonian translocation between chromosomes 5B and 7B in Arina*LrFor*. **f**, **g**, Cytology (**f**) and Hi-C (**g**) confirm the 5B/7B translocation in SY Mattis (left) compared with the non-carrier Norin 61 (right). In **f**, five independent cells were observed; the translocation was confirmed independently ten times. Scale bar, 10 μm.

**Extended Data Fig. 5:**
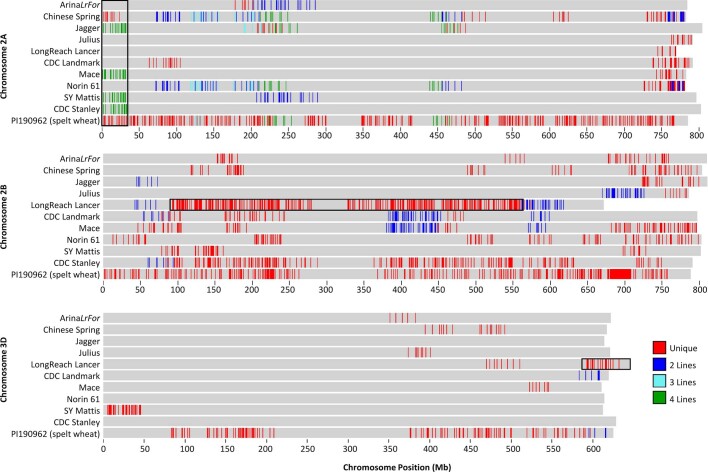
Identification of alien introgressions from wheat relatives. A feature of foreign chromosomal introgressions is that they contain unique patterns of TE insertions. Shown are stretches of >20 Mb containing multiple polymorphic RLC-*Angela* retrotransposons that are found only in one or a few (≤4) of the sequenced lines. One representative chromosome for each wheat subgenome is shown. Individual polymorphic retrotransposons are indicated as coloured vertical lines. Colours correspond to the number of cultivars a foreign segment is found in. Regions of particular interest are indicated by black rectangles. These include the 2N^v^S alien introgression from *A. ventricosa* at the end of chromosome 2A in Jagger, Mace, SY Mattis and CDC Stanley, as well as introgression in the central region of chromosome 2B from *T. timopheevi* in LongReach Lancer, and introgression at the end of chromosome 3D from *T. ponticum* in LongReach Lancer.

Transposable elements make up a large majority of the wheat genome and have a critical role in genome structure and gene regulation. We characterized the overall transposable element content (81.6%) and its composition (69% long terminal-repeat retrotransposons (LTR) and 12.5% DNA transposons) in the RQAs (Supplementary Table 5). Across all RQAs, we annotated 1.22 × 10^6^ full length (fl)-LTRs, which clustered lines into the same groups we observed from our analysis of PAV and SNPs (Fig. 1a, b, Extended Data Fig. 3d). Generally, unique fl-LTRs (147,450) were young (median of 0.9 million years) and were enriched in the highly recombining, more distal chromosomal regions (Fig. 1d). By contrast, shared fl-LTRs were older (median of 1.3 million years) and were more evenly distributed across the pericentric regions (Fig. 1d). The RLC-*Angela* fl-LTRs were the most abundant (21,000–27,000 full-length copies per genome) and analysis of variant patterns identified several chromosomal segments that contained numerous unique or rare retrotransposon insertions (Extended Data Fig. 5), which, on the basis of breeding history, we hypothesize to represent introgressions. For example, the LongReach Lancer RQA revealed two unique regions, a pericentric region on chromosome 2B and a segment on the end of chromosome 3D (Fig. 2a, b), both of which affect chromosome length (Extended Data Fig. 5). We used pedigree analysis to postulate the source of the introgressions and performed whole-genome sequencing of multiple accessions of putative donors. LongReach Lancer carries the stem rust resistance gene *Sr36*, derived from an introgression from *Triticum timopheevii*, and the resistance genes *Lr24* (leaf rust) and *Sr24* (stem rust), derived from tall wheatgrass^28,29^ (*Thinopyrum ponticum*). We generated whole-genome sequence reads from multiple *T. ponticum* and *T. timopheevii* accessions (Supplementary Table 12) and alignment to the LongReach Lancer RQA confirmed a *T. ponticum* introgression spanning a region of approximately 60 Mb of chromosome 3D (Fig. 2a), whereas *T. timopheevii* aligned to the majority (427 Mb) of chromosome 2B (Fig. 2b). Overall, we identified 341 chromosomal segments larger than 20 Mb with unique or rare fl-LTR insertion patterns that were present in only 1 to 4 of the RQA genomes, of which 273 insertion patterns were uniquely associated with a single genome (Supplementary Tables 13–16). The majority of unique regions were in PI190962 (spelt wheat; *Triticum aestivum* ssp. *spelta*), which was expected, given that it diverged from modern bread wheat several thousand years ago.

**Extended Data Fig. 6:**
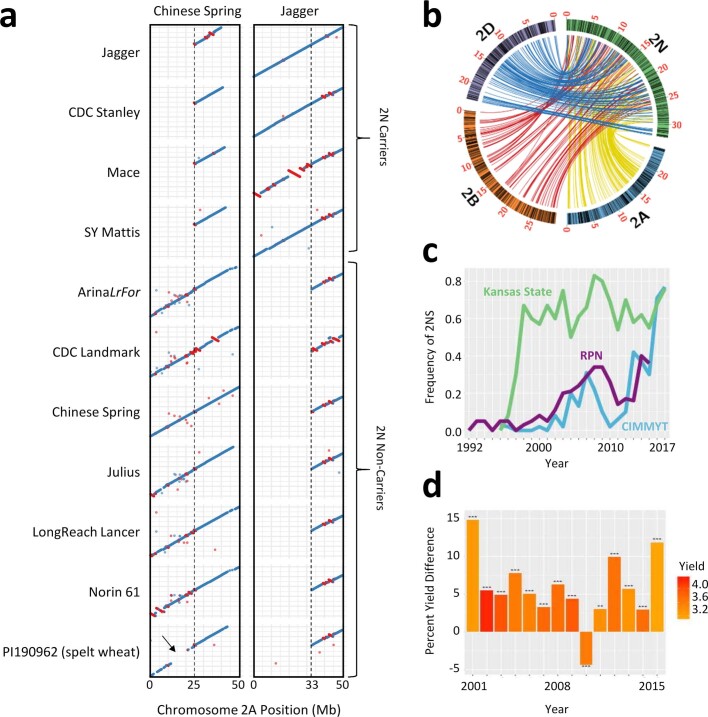
Detailed characterization of the 2N^v^S introgression from *A. ventricosa*. **a**, Pairwise alignments of the first 50 Mb of chromosome 2A. The black arrow indicates a possible unique haplotype within spelt. **b**, Orthologous genes between the 2N^v^S introgression from *A. ventricosa* in Jagger and the genes on chromosomes 2A, 2B, and 2D in Chinese Spring. **c**, Frequency of 2N^v^S introgression carriers in North American datasets from CIMMYT, Kansas State, and the USDA Winter Wheat Regional Performance Nursery (RPN) over time. **d**, Per cent yield difference in lines that carry the 2N^v^S introgression. Two sided *t*-tests were performed to test for the significance of the impact of the 2N^v^S introgression. **P < 0.01; ***P < 0.001.

A similar strategy was used to confirm RLC-*Angela* variation at the telomeric region of chromosome 2A in Jagger, Mace, SY Mattis and CDC Stanley (Fig. 2c), which corresponds to the 2NvS introgression from *Aegilops ventricosa* (Supplementary Note 5). This introgression is a well-known source of resistance to wheat blast^30^, and contains the *Lr37–Yr17–Sr38* gene cluster, which provides resistance to several rust diseases^31^. Sequencing of *A. ventricosa* accessions (Supplementary Table 12) followed by comparison of chromosomes with the RQAs confirmed that Jagger, Mace, SY Mattis and CDC Stanley carry the 2NvS introgression, which spans about 33 Mb on chromosome 2A (Fig. 2c, Extended Data Fig. 6a). We annotated the coding genes within this region and identified 535 high-confidence genes; more than 10% were predicted to be associated with disease resistance, including genes that encode putative NB-ARC and NLRs (Extended Data Fig. 6b, Supplementary Tables 17, 18). Furthermore, we used genotyping by sequencing to detect the 2NvS segment in three wheat panels and discovered that its frequency has been increasing in breeding germplasm and its presence is consistently associated with higher grain yield (Extended Data Fig. 6c, d, Supplementary Tables 19, 20). Of note, we identified about 60 genes belonging to the cytochrome P450 superfamily, which have been implicated in abiotic and biotic stress tolerance^32^ and have been functionally validated to influence grain yield in wheat^33^. Together, these data indicate that the modern wheat gene pool contains many chromosomal segments of diverse ancestral origins, which can be identified by their transposable-element signatures. We also confirmed the wild-relative origins of three introgressions within the RQA assemblies—a first step towards characterizing causal genes for breeding targets, such as resistance to wheat blast and rust fungi.

## Centromere dynamics

**Extended Data Fig. 7:**
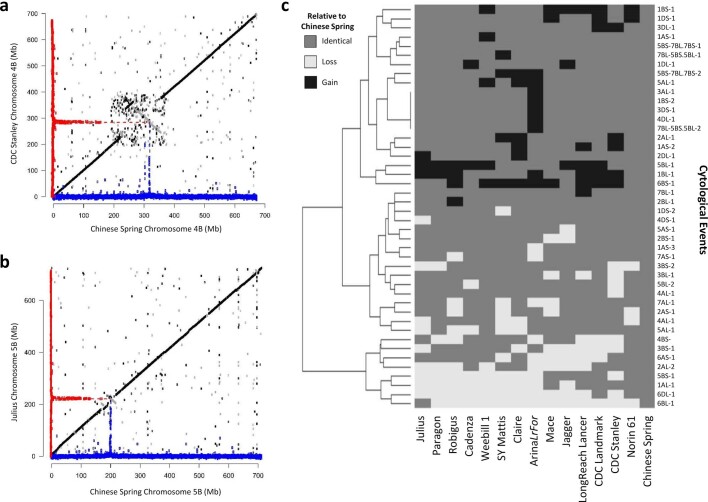
Centromere positions and karyotype variation. Functional centromere positions in the RQA have undergone structural and positional rearrangement. Chromosome alignments showing collinearity (black scaffolds in same orientation, grey scaffolds in opposite orientation) with relative density of CENH3 ChIP–seq mapped to 100 kb genomic bins for Chinese Spring (blue) and a representative genome of comparison (red) for chromosome 4B of CDC Stanley (**a**), and chromosome 5B of Julius (**b**). **c**, Detailed list and clustering of cytological features carried by each wheat line (Supplementary Note 6). Features that are identical (dark grey) or have a gain (black) or loss (light grey) relative to Chinese Spring are indicated.

Centromeres are vital for cell division and chromosome pairing during meiosis. In plants, functional centromeres are defined by the epigenetic placement of the modified histone CENH3^34^. We therefore used CENH3 chromatin immunoprecipitation and sequencing (ChIP–seq)^35^ to determine the positions and sizes (about 7.5–9.6 Mb) of the centromeres for each RQA (Supplementary Tables 21, 22), which were consistent with previous estimates for wheat^1^. Furthermore, all chromosomes showed a single active site, implying that previous reports of multiple active centromeres in Chinese Spring^1^ were artefacts of misoriented scaffolds. However, we found examples in which the relative position of the centromere was shifted owing to several pericentric inversions, including inversions on chromosomes 4B and 5B (Extended Data Fig. 7a, b). We also observed one instance in which the centromeric position changed, but was not associated with a structural event. Specifically, on chromosome 4D in Chinese Spring, the centromere is shifted by around 25 Mb relative to the consensus position (Fig. 2d). This shift was previously recognized by cytology but was hypothesized to result from a pericentric inversion^36^. However, the high degree of collinearity between genomes supports the hypothesis that Cen4D in Chinese Spring has shifted to a non-homologous position; this shifting of centromeres to non-homologous sites has also been reported in maize^37^. By characterizing the centromere positions for these diverse wheat lines, we provide strong evidence for changes in centromere position caused by structural rearrangements and centromere shifts.

## Large-scale structural variation between genomes

**Extended Data Fig. 8:**
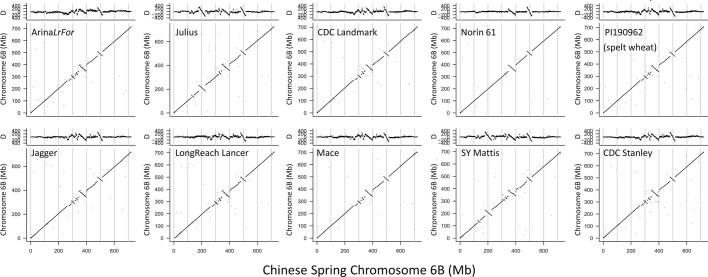
Hi-C validates inversions identified from pairwise chromosome alignments. Pairwise alignments of chromosome 6B from the RQA and Chinese Spring are shown. Above each alignment dot plot, the directionality biases estimated from alignments of Hi-C data against Chinese Spring are shown. Boundaries of diagonal segments are indicative of inversions and coincide with inversion boundaries identified from the chromosome alignments.

**Extended Data Fig. 9:**
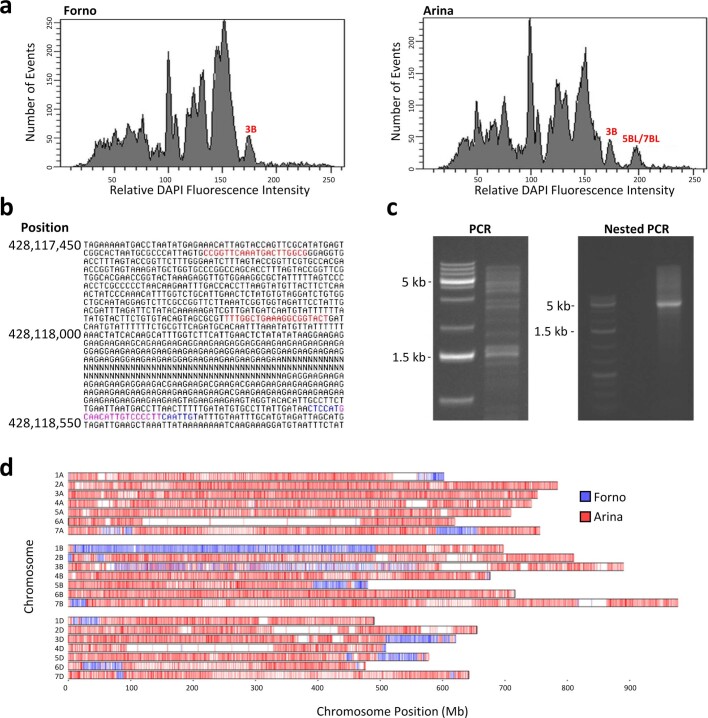
Characterization of a translocation involving wheat chromosomes 5B and 7B. **a**, Cytogenetic karyotypes of Forno (left) and Arina (right), the parents of Arina*LrFor*. Note that the large recombinant chromosome 7B is represented by a distinct peak. **b**, Sequence of the translocation breakpoint on chromosome 7B of Arina*LrFor*. Note that the exact breakpoint lies in a sequence gap (stretch of Ns). The bp positions are indicated at the left. Forward PCR primers are shown in red and reverse primers in blue. The overlap of the two reverse primers is shown in purple. The outer primer pair was used for PCR, while the inner pair was used for a nested PCR. **c**, PCR amplification of the fragment spanning the translocation breakpoint. The nested PCR yielded a ~5 kb fragment that spanned the translocation breakpoint and its identity was confirmed by sequencing. Both PCR and nested PCR were performed in duplicate; both replicates of the nested PCR were sequenced using the Sanger method. For gel source data, see Supplementary Fig. 1. **d**, Mapping of Illumina reads from the cultivars Arina and Forno on to the pseudomolecules of Arina*LrFor*. Sequence derived from Forno is shown in blue, while sequenced derived from Arina is in red. Note that chromosomes 5B and 7B are derived from both parents, indicating that these parental chromosomes can recombine freely, despite the presence of a large 5B/7B translocation in Arina.

Structural variants are common in wheat^38^, and impact genome structure and gene content. We characterized large structural variants using pairwise genome alignments (Extended Data Fig. 1), changes in three-dimensional topology of chromosomes revealed by Hi-C conformation capture directionality biases along the genome^39,40^ (Extended Data Fig. 8, Supplementary Table 23), which were confirmed by Oxford Nanopore long-read sequencing (Extended Data Fig. 2) and cytological karyotyping (Extended Data Fig. 7c, Supplementary Table 24, Supplementary Note 6). The most prominent event was a translocation between chromosomes 5B and 7B, observed in Arina*LrFor*, SY Mattis (Fig. 2e–g) and Claire. Normally, chromosomes 5B and 7B are approximately 737 and 762 Mb long, respectively, and we estimated that the recombined chromosomes are 488 Mb (5BS/7BS) and 993 Mb (7BL/5BL) long, making 7BL/5BL the largest wheat chromosome (Extended Data Fig. 9a). In Arina*LrFor* and SY Mattis, the 7BL/5BL breakpoint resides within an approximately 5-kb GAA microsatellite, which we were able to span using polymerase chain reaction (PCR) (Extended Data Fig. 9b, c). By contrast, the breakpoint on 5BS/7BS was less syntenic, and we detected polymorphic fluorescence in situ hybridization signals between Arina*LrFor* and SY Mattis on the 5BS portion of the translocated chromosome segment, suggesting that the regions adjacent to the translocation events differ on 5BS/7BS (Supplementary Note 6). To determine the stability of the translocation in breeding, we genotyped for the translocation event in a panel of 538 wheat lines that represent most of the UK wheat gene pool grown since the 1920s^41^. The translocation occurred in 66% of the lines and was selectively neutral (Supplementary Note 7). Notably, the *Ph1* locus on chromosome 5B, which controls the pairing of homeologous chromosomes during meiosis^42^, is near the translocation breakpoint, but remained highly syntenic between translocation carriers and non-carriers. Genetic mapping and analysis of short-read sequencing data indicated that the 5B/7B translocated chromosomes recombine freely with 5B and 7B chromosomes (Extended Data Fig. 9d), suggesting that chromosome pairing is not affected by the translocation.

## Haplotype-based gene mapping

**Fig. 3:**
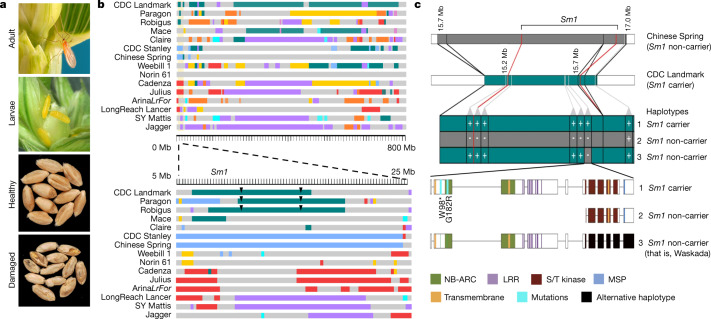
Cloning of the gene *Sm1*. **a**, The orange wheat blossom midge oviposits eggs on wheat spikes and the larvae feed on developing wheat grains, resulting in moderate to severe damage to mature kernels. **b**, Top, sections of chromosome 2B of the same colour in the same position share haplotypes (based on 5-Mb bins), with the exception of those in grey, which indicates a line-specific haplotype. The position of *Sm1* is indicated with respect to the CDC Landmark assembly. Bottom, zoomed-in view of haplotype blocks (based on 250-kb bins) from 5 to 25 Mb positions on chromosome 2B, surrounding *Sm1*. CDC Landmark, Robigus and Paragon all carry the same haplotype surrounding *Sm1* (teal). **c**, Top, anchoring of the *Sm1* fine map to the physical maps of Chinese Spring and CDC Landmark and graphical genotypes of three haplotypes critical to localizing the *Sm1* candidate gene. Bottom, annotation of the *Sm1* candidate gene, which encodes NB-ARC and LRR motifs in addition to the integrated serine/threonine (S/T) kinase and MSP domains. Two independent ethyl-methanesulfonate-induced mutations (W98* and G182R) result in loss of function and susceptibility to the orange wheat blossom midge (light blue lines). An alternative haplotype was observed in the kinase region of Waskada (black).

**Extended Data Fig. 10:**
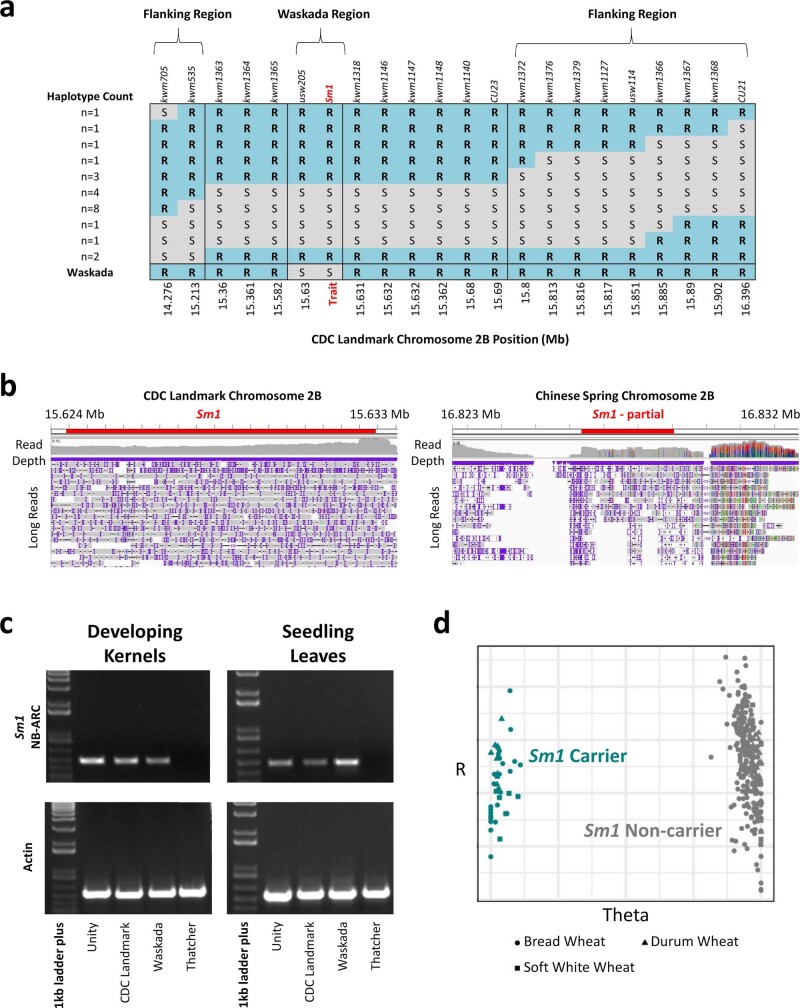
Confirmation of gene expression and gene structure for *Sm1*. **a**, Critical recombinants from the 99B60-EJ2G/Infinity and 99B60-EJ2D/Thatcher populations used to fine map *Sm1*. The 99B60-EJ2G/Infinity cross had 5,170 F_2_ plants, while 99B60-EJ2D/Thatcher cross had 5,264 F_2_ plants; only recombinant haplotypes between orange wheat blossom midge resistant (R) and susceptible (S) genotypes are shown. **b**, Oxford Nanopore long read confirmation of the *Sm1* gene candidate in the CDC Landmark RQA (left), and alternative haplotype in Chinese Spring (right). Vertical coloured lines indicate sequence variants. **c**, Amplification of cDNA for the NB-ARC domain of the *Sm1* gene candidate (top) and actin control (bottom) derived from RNA isolated from developing kernels (left) and wheat seedlings (right). Unity and CDC Landmark are carriers of *Sm1*. Waskada carries an alternative haplotype and does not carry *Sm1* (see main text). Thatcher was used as a susceptible parent for fine mapping of *Sm1* and does not contain the associated NB-ARC domain. The experiment was replicated on four independent biological samples for each condition. **d**, Distribution of an *Sm1* allele-specific PCR marker in a diverse panel of >300 wheat lines.

To develop improved wheat cultivars, breeders shuffle allelic variants by making targeted crosses and exploiting the recombination that occurs during meiosis. These alleles, however, are not inherited independently, but rather as haplotype blocks that often extend across multiple genes that are in genetic linkage^43,44^. We quantified haplotype variation along chromosomes across the assemblies, and developed visualization software to support its utility (Supplementary Note 8). We used these haplotypes to characterize a locus that provides resistance to the orange wheat blossom midge (OWBM, *Sitodiplosis mosellana* Géhin), one of the most damaging insect pests of wheat, which is endemic in Europe, North America, west Asia and the Far East. Upon hatching, the first-instar larvae feed on the developing grains and damage the kernels (Fig. 3a). *Sm1* is the only gene in wheat known to provide resistance to OWBM^6^. CDC Landmark, Robigus and Paragon are all resistant to the OWBM, and all three carry the same 7.3-Mb haplotype within the *Sm1* locus on chromosome 2B (Fig. 3b). To identify *Sm1* gene candidates, we used high-resolution genetic mapping and refined the locus to a 587-kb interval in the CDC Landmark RQA (Fig. 3c, Extended Data Fig. 10a, Supplementary Table 25). Through extensive genotyping of diverse breeding lines, we found an OWBM-susceptible line, Waskada, that displayed a resistant haplotype except near one gene, which we annotated in CDC Landmark to encode a canonical NLR with kinase and major sperm protein (MSP) integrated domains (Fig. 3c). Oxford Nanopore long-read sequencing further confirmed the structure of the gene in CDC Landmark (Extended Data Fig. 10b). By contrast, the remaining assemblies (susceptible to OWBM) lacked the NB-ARC domain, but the kinase and MSP domains remained intact (Fig. 3c). We sequenced the Waskada allele and found it contains the NB-ARC domain, but an alternative haplotype within the kinase domain (Fig. 3c, Extended Data Fig. 10c). This gene is expressed in wheat kernels and seedlings of *Sm1* carrier lines, and the lack of cDNA amplification of the NB-ARC domain for non-carrier lines further supported an alternative gene structure (Extended Data Fig. 10c). We generated two knockout-mutant lines of this candidate gene in the *Sm1* carrier line Unity^45^, and both were consistently rated as susceptible to OWBM (Supplementary Table 26). Sequencing of the candidate gene in these two mutants revealed a single point mutation in each line: a G>A mutation resulting in a Gly>Arg (G182R) amino acid substitution in the NB-ARC domain, and a G>A mutation, resulting in a stop codon (W98*) before the NB-ARC domain (Fig. 3c). The kinase domain encoded by *Sm1* belongs to the serine/threonine class^46^, similar to those of *Rpg5*, which provides stem rust resistance^47^, and *Tsn1*, which encodes sensitivity to the necrotrophic effector ToxA produced by *Parastagonospora nodorum* and *Pyrenophora tritici-repentis*^48^; however, both *Rpg5* and *Tsn1* lack the MSP domain. To our knowledge, this is the first report of an NB-ARC-LRR-kinase-MSP coding gene associated with insect resistance. Additional research is needed to functionally validate these domains and their putative role in OWBM resistance using tools such as gene editing. Nevertheless, we developed a high-throughput and low-cost competitive allele-specific PCR marker (KASP) that discriminates between OWBM-susceptible and OWBM-resistant lines with perfect accuracy (Extended Data Fig. 10d, Supplementary Table 27). Our analyses, along with the haplotype and synteny viewers (https://kiranbandi.github.io/10wheatgenomes/, http://10wheatgenomes.plantinformatics.io/ and http://www.crop-haplotypes.com/), laid the foundation for identifying haplotypes for *Sm1*. Haplotypes can now be genotyped in breeding programs using single-marker or high-throughput-sequencing-based approaches, which can integrate desirable genes into improved cultivars more efficiently.

## Discussion

We have built on the genome-sequence resources available for wheat and related species to produce ten RQAs and five scaffolded assemblies that represent hexaploid wheat lines from different regions, growth habits and breeding programs^1,11,12,18,20,49^. We have identified and characterized SNPs, PAV, CNV, centromere shifts, large-scale structural variants and introgressions from wild relatives of wheat that can be used to identify and characterize important breeding targets. This was complemented by a transposable-element-analysis approach to identify candidate introgressions from wild relatives of wheat, for which we provided high-quality assemblies of segments already used in global breeding programs. Together, these RQAs present an opportunity for breeders and researchers to perform high-resolution manipulation of genomic segments and pave the way to identifying genes responsible for in-demand traits, as we demonstrated for resistance to the insect pest OWBM. Functional gene studies will also be facilitated by comparative gene analyses, as exemplified by our analyses of orthologous groups, *Rf* genes and NLR immune receptors^26^. Finally, we highlight haplotype blocks, which will facilitate marker development for applied breeding^43,50^. Equipped with multiple layers of data describing variation in wheat, we now have powerful tools to increase the rate of wheat improvement to meet future food demands.

## Methods

No statistical methods were used to predetermine sample size. The field experiments were randomized, but the wheat lines sequenced and assembled were not selected at random. The investigators were not blinded to allocation during experiments and outcome assessment.

### Assemblies and annotation

#### Genome assemblies

We assembled the genomes of 15 diverse wheat lines using two approaches (Supplementary Table 1). The RQA approach used the DeNovoMAGIC v.3.0 assembly pipeline, previously used for the wild emmer wheat^11^, durum wheat^12^ and Chinese Spring RefSeqv1.0 assemblies. In brief, high-molecular-weight DNA was extracted from wheat seedlings as described previously^51^. Illumina 450-bp paired-end (PE), 800-bp PE and mate-pair (MP) libraries of three different sizes (3 kb, 6 kb and 9 kb) were generated. Sequencing was performed at the University of Illinois Roy J. Carver Biotechnology Center. 10X Genomics Chromium libraries were prepared and sequenced at the Genome Canada Genome Innovation Centre using the manufacturers’ recommendations to achieve a minimum of 30 × coverage. Hi-C libraries were prepared using previously described methods^40^. Using the Illumina PE, MP, 10X Genomics Chromium, and Hi-C, chromosome scale assemblies were prepared as described previously^18^. For cultivars assembled to a scaffold level, we used the W2RAP-contigger using *k* = 200 (Supplementary Note 1). Two MP libraries (10 kb and 13 kb) were produced for each line except Weebill 1, for which two additional MP libraries were used. Mate pairs were processed, filtered and used to scaffold contigs as described in the W2RAP pipeline (https://github.com/bioinfologics/w2rap). Scaffolds of less than 500 bp were removed from the final assemblies. Additionally, we performed Oxford Nanopore sequencing of CDC Landmark using R9 flow cells and the GridION sequencing technology (Supplementary Note 2).

#### Nucleotide diversity analysis

The variant call format data files from two wheat exome-capture studies^4,5^ were retrieved, combined, and filtered to retain hexaploid accessions and polymorphisms detected in both studies. The 10X Genomics Chromium sequencing data for each of the RQA lines were aligned to Chinese Spring RefSeqv1.0 using the LongRanger v.2.1.6 software. Alignment files from the accessions assembled here and 16 Bioplatforms Australia lines^19^ with alignments obtained from the DAWN project^52^ were then used for variant calling by GATK v.3.8 at the same genomic positions identified by exome-capture sequencing. The variant files from the exome-capture studies, DAWN project and 10+ Wheat Genomes lines were then merged and subjected to principal component analysis (PCA) using the prcomp function in R v.3.6.1.

#### Gene projections

We used the previously published high-confidence gene models for Chinese Spring to assess the gene content in each assembly. Representative coding sequences of each informant locus were aligned to pseudomolecules of each line separately using BLAT^53^ v.3.5 with the ‘fine’ parameter and a maximal intron size of 70 kb. BLAT matches seeded an additional alignment by exonerate^54^ in the genomic neighbourhood encompassing 20 kb upstream and downstream of the match position. Exonerate alignments required a minimal and maximal intron sizes of 30 bp and 20 kb, respectively. A linear regression of colocalized matches with complete alignments of the informant were computed for 10,000 such pairs to derive a normalization function and to render comparable scoring schemes for both methods. Subsequently, we selected the top-scoring match for each mapping pair as the locus for the gene projection. Projections were then filtered by alignment coverage (Supplementary Note 3), the open reading frame (ORF) contiguity, the observed mapping frequency of the informant, coverage of start and stop codons, and the orthology or potential dislocation of the match scaffold relative to its informant chromosome. Identification of orthologous groups was analogous to the approach used previously^55^. Reciprocal best BLAST hit (RBH) graphs were derived from pairwise all-against-all BLASTn v2.8 transcript searches (minimal *e*-value ≤ 1 × 10^−30^). Hits were assigned to homeologous groups on the basis of gene models of Chinese Spring following a previously described homeologue classification^9^. Multiple sequence alignments for the population genetics analysis were performed using MUSCLE v.3.8 with default parameters (Supplementary Note 3). Using the gene projections, we quantified average pairwise genetic diversity (*π*), polymorphism (Watterson’s *θ*_W_), and Tajima’s *D* using compute and polydNdS in the libsequence v.1.0.3-1 package^56^. We retained diversity estimates for genes that were in all of the genomes and had ≤100 segregating sites. PAV was determined from the orthologous groups limited to one-to-one relations where there was no match in at least one genome.

#### Analysis of the *Rf*-like gene family

For *Rf* genes, the genome sequences were scanned for ORFs in six frame translations with the getorf program of the EMBOSS v.6.6.0 package. ORFs longer than 89 codons were searched for the presence of PPR motifs using hmmsearch from the HMMER v.3.2.1 package (http://hmmer.org) and the hidden Markov models defined previously. The PF02536 profile from the Pfam v32.0 database (http://pfam.xfam.org) was used to screen for ORFs carrying mTERF motifs. Downstream processing of the hmmsearch results followed the pipeline described previously^57^. ORFs with low hmmsearch scores were removed from the analysis as they are unlikely to represent functional PPR proteins. Only genes encoding mTERF proteins longer than 100 amino acids were included in the analysis. *RFL*-PPR sequences were identified as described^23^. The phylogenetic analyses were performed as described previously^23^. Conserved, non-PPR genes delimiting the borders of analysed *RFL* clusters were identified in the Chinese Spring RefSeqv1.0 reference genome and used to search for syntenic regions in the remaining wheat accessions with BLAST v.2.8. See Supplementary Note 4 for more details.

#### NLR repertoire

NLR signatures were annotated using NLR-Annotator^58,59^ (https://github.com/steuernb/NLR-Annotator) with the option -a. We estimated redundancy of NLR signatures between genomes at different thresholds of identity: 95%, 98% and 100%. For the 165 amino acids in the consensus of all NB-ARC motifs, this translates to 8, 3 and 0 mismatches of a concatenated motif sequence. To calculate the overall redundancy in all genomes, we counted the number of LR signatures added to a non-redundant set by adding genomes iteratively. This was done for 1 million random permutations.

#### Repeat annotation

Transposons were detected and classified by a homology search against the REdat_9.7_Poaceae section of the PGSB transposon library^60^ using vmatch (http://www.vmatch.de) with the following parameters: identity ≥70%, minimal hit length 75 bp, seedlength 12 bp (exact command line: -d -p -l 75 -identity 70 -seedlength 12 -exdrop 5). To remove overlapping annotations, the output was filtered for redundant hits via a priority-based approach in which higher-scoring matches where assigned first and lower-scoring hits at overlapping positions were either shortened or removed if there was ≥90% overlap with a priority hit or if <50 bp remained. Tandem repeats where identified with TandemRepeatFinder v.4.09 under default parameters^61^ and subjected to overlap removal as described above. Full-length LTR retrotransposons were identified with LTRharvest (http://genometools.org/documents/ltrharvest.pdf). All candidates were subsequently annotated for PfamA domains using HMMER v.3.0 and filtered to remove false positives, non-canonical hybrids and gene-containing elements. The inner domain order served as a criterion for the LTR retrotransposon superfamily classification, either Gypsy (RLG: RT-RH-INT), Copia (RLC: INT-RT-RH) or undetermined (RLX). The insertion age of fl-LTRs was calculated from the divergence between the 5′ and 3′ long terminal repeats, which are identical upon insertion. The genetic distance was calculated with EMBOSS v.6.6.0 distmat (Kimura2-parameter correction) using a random mutation rate of 1.3 × 10^−8^.

#### Analysis of centromeric regions

For each line with a RQA, ChIP was performed according to previous methods^62^ with slight modification using a wheat-specific CENH3 antibody^36^. An antigen with the peptide sequence RTKHPAVRKTKALPKK, corresponding to the N terminus of wheat CENH3, was used to produce an antibody using the custom-antibody production facility provided by Thermo Fisher Scientific. The customized antibody was purified and obtained as pellets. The antibody pellet (0.396 mg) was dissolved in 2 ml PBS buffer, pH 7.4, resulting in a working concentration of 198 ng μl^−1^. Nuclei were isolated from 2-week-old seedlings, digested with micrococcal nuclease and incubated overnight at 4 °C with 3 μg of antibody or rabbit serum (control). Antibodies were captured using Dynabeads Protein G and the chromatin eluted using 100 μl of 1% sodium dodecyl sulfate, 0.1 M NaHCO_3_ preheated to 65 °C. DNA isolation was then performed using ChIP DNA Clean & Concentrator Kit, and ChIP–seq libraries were constructed using TruSeq ChIP Library Preparation Kit and sequenced with a NovoSeq S4, which generated 150-bp paired-end reads.

For Chinese Spring, we used two datasets, SRR1686799^63^ (dataset 1) and the dataset generated in this study (dataset 2). Sequence reads were de-multiplexed, trimmed and aligned to each of the respective RQAs using HISAT2 v.2.1.0^64^. Alignments were sorted, filtered for minimum alignment quality of 30, counted in 100-kb bins using samtools v.1.10 and BEDtools v.2.29, and visualized in R v.3.6.1. To define the midpoint of each centromere, we identified the highest density of CENH3 ChIP–seq reads using a smoothing spline in R v.3.6.1 with smooth.spline function (number of knots = 1,000) and identified the peak of the smooth spline as the centre of the respective centromere for a given chromosome. To compare centromeric positions of different genomes, the CENH3 ChIP–seq density was plotted along with MUMmer v.4.0 chromosome alignments. To determine the overall size of wheat centromeres, we considered each 100-kb bin with CENH3 ChIP–seq read density that was greater than three times the background (genome average) level of read density to be an active centromeric bin. The number of enriched bins for each genome were counted and averaged to a total of 21 chromosomes. This calculation included counting of unanchored bins.

### Analysis of introgressions

#### Identification of full-length RLC-*Angela* retrotransposons

Retrotransposon profiles were created for each genome using the RLC-*Angela* family^65^ and consensus sequences obtained from the TREP database (www.botinst.uzh.ch/en/research/genetics/thomasWicker/trep-db.html). First, BLASTn was used to compare the ~1,700-bp LTR of RLC-*Angela* to each genome. Matching elements and 500 bp of flanking sequences were aligned to identify precise LTR borders as well as different sub-families and/or sequences variants. We then used BLASTn to compare the 18 consensus LTR sequences against each genome and then screened for pairs of full-length LTRs that are found in the same orientation within a window of 7.5–9.5 kb (RLC-*Angela* elements are ~8.7 kb long). These initial candidate full-length elements were screened for the presence of RLC-*Angela* polyprotein sequences by BLASTx, as well as for the typical 5-bp target-site duplications. We allowed a maximum of two mismatches between the two target-site duplications. All identified full-length RLC-*Angela* copies were then aligned to a RLC-*Angela* consensus sequence with the program Water from the EMBOSS v.6.6.0 package (www.ebi.ac.uk/Tools/emboss/). These alignments were used to compile all nucleotide polymorphisms into a single file. The variant call file was then used for PCA using the snpgdsPCA function in the R package SNPrelate v.3.11.

#### Sequencing of the tertiary gene pool of wheat

Genomic DNA (gDNA) was extracted and purified from young leaf tissue collected from multiple accessions of *T. timopheevii*, *A. ventricosa* and *T. ponticum* (Supplementary Table 12) following a standard CTAB–chloroform extraction method. Yield and integrity were evaluated by fluorometry (Qubit 2.0) and agarose gel electrophoresis. Paired-end libraries were prepared following the Nextera DNA Flex protocol. In brief, 500 ng gDNA from each accession was fragmented and amplified with a limited-cycle PCR. Each library was uniquely dual-indexed with a distinct 10-bp index code (IDT for Illumina Nextera DNA UD) for multiplexing, and quantified by qPCR (Kapa Biosystems). Final average library size was estimated on a Tapestation 2200. Libraries were normalized and pooled for sequencing on an Illumina NovaSeq 6000 S4 to generate ~5× coverage per genotype. Sequencing data were de-multiplexed and aligned to appropriate RQAs (Supplementary Table 12) in semi-perfect mode using the BBMap v.38 short-read alignment software (https://sourceforge.net/projects/bbmap/).

### Structural variation

We karyotyped the lines using mitotic metaphase chromosomes prepared by the conventional acetocarmine-squash method. Non-denaturing fluorescence in situ hybridization (ND-FISH) of three repetitive sequence probes, Oligo-pSc119.2-1, Oligo-pTa535 and Oligo-pTa713, was performed as described^66,67^ (Supplementary Note 6). Chromosomes were counterstained with DAPI. Chromosome images were captured with an Olympus BX61 epifluorescence microscope and a CCD camera DP80. Images were processed and pseudocoloured with ImageJ v.1.51n in the Fiji package. For karyotyping, at least four chromosomes per accession were examined and compared to the karyotype of Chinese Spring as described previously^68^. Hierarchal clustering of karyotype polymorphisms was performed using the Ward method in R v.3.0.2, which was used to estimate distance. Next, we applied Hi-C analysis for inversion calling as described previously^40^. In brief, adapters were removed and reads were mapped to Chinese Spring using minimap2 v.2.10^69^ as we have done previously^21^. The raw Hi-C link counts were calculated in 1 Mb non-overlapping sliding windows and then normalized as described in our previous work^40^. Finally, the normalized Hi-C link matrix was subjected to inversion calling using R.

We performed flow cytometry of wheat cultivars Arina and Forno as previously described^70^, except that we used a FACSAria SORP flow cytometer and cell sorter (Becton Dickinson). The 5B/7B translocation breakpoints were identified by comparison of chromosomes 5B and 7B from Arina*LrFor* and Julius. Sequence collinearity between Arina*LrFor* and Julius was detected by BLASTn searches of 1,000-bp sequence windows every 100 kb along the chromosomes. Once an interruption of synteny was detected, sequence segments at the positions of synteny loss were extracted and used for local alignments to determine the precise breakpoint positions. PCR amplification of the 5BS/7BS and 7BL/5BL translocation sites was performed using standard PCR cycling conditions.

### Characterization of haplotypes

#### Development of a wheat genome haplotype database

To identify haplotypes, pairwise chromosome alignments were performed between the RQA using MUMmer v.4.0, which were combined with pairwise nucleotide BLASTn analyses of the genes ± 2,000 bp using custom scripts in R v.3.6.1 (https://github.com/Uauy-Lab/pangenome-haplotypes)^71^ (Supplementary Note 8). The resultant haplotypes were uploaded to an interactive viewer (http://www.crop-haplotypes.com/). Pairwise BLASTn comparisons of the genes were also used to identify structural variants, and were uploaded into AccuSyn (https://accusyn.usask.ca/) and SynVisio (https://synvisio.github.io/#/) to create a wheat-specific database (https://kiranbandi.github.io/10wheatgenomes/). Pretzel (https://github.com/plantinformatics/pretzel) was also used to visualize and compare the RQA and the projected gene annotations (http://10wheatgenomes.plantinformatics.io/).

#### Characterization of *Sm1*

*Sm1*-linked markers^6^ were located in RQAs using BLAST v.2.8.0. Two high-resolution mapping populations were developed, 99B60-EJ2D/Thatcher and 99B60-EJ2G/Infinity. Progeny heterozygous for crossover events near *Sm1* were identified in the F_2_ generation, and the crossovers were fixed in the F_3_ generation. The resulting F_2_-derived F_3_ families were analysed with KASP markers within the *Sm1* region and tested for resistance to OWBM in field nurseries to identify markers associated with *Sm1*. Ethyl methanesulfonate was used to develop knockout mutants in the *Sm1* gene. Approximately 3,200 seeds of the Canadian spring wheat variety Unity (an *Sm1* carrier) were soaked in a 0.2% (v/v) aqueous ethyl methanesulfonate solution for 22 h at 22 °C. The seed was then rinsed in distilled water and sown in a field nursery. The M_1_ seed was grown to maturity and bulk harvested. Approximately 6,000 M_2_ seeds were space planted in two field nurseries located in Brandon and Glenlea, Manitoba, Canada. Spikes were collected on a per-plant basis at maturity and were classified as resistant, susceptible or undamaged as done previously^6,72^. Putative *Sm1*-knockout mutants were re-tested for OWBM resistance in indoor cage tests^73^ in the M_3_ and M_4_ generations. M_4_-derived families were tested for resistance to OWBM in field nurseries (randomized complete block design, six environments, and eight replicates per environment).

Candidate genes were identified between *Sm1* flanking markers on the CDC Landmark assembly using the projected gene annotations and FGENESH v.2.6 (http://www.softberry.com/), which were compared to the projected genes of non-carriers. Both 5′ and 3′ rapid amplification of cDNA ends (5′ and 3′ RACE) were used to verify the transcription initiation and termination sites of the gene candidate, whose structure was predicted by FGENESH v.2.6. In brief, RNA was extracted from the leaves of Unity (*Sm1* carrier) seedlings (using the Qiagen RNeasy kit), RACE PCR performed (Invitrogen GeneRacer kit), and the PCR product cloned (Invitrogen TOPO TA Cloning kit for sequencing) and sequenced by Sanger sequencing. Prediction of the conserved domains was done using the NCBI Conserved Domain Search tool (https://www.ncbi.nlm.nih.gov/Structure/cdd/wrpsb.cgi) and PROSITE (release 2020_01; https://prosite.expasy.org/). The LRR domain was defined on the basis of the presence of 2–42 LRR motif repeats of 20–30 amino acids each. LRR motifs were manually annotated^74^. Prediction of transmembrane regions and orientation was performed using the program TMpred NCBI Conserved Domain Search tool (https://embnet.vital-it.ch/software/TMPRED_form.html).

To study the expression of *Sm1*, total RNA was extracted from four biological replicates from four wheat genotypes (Unity, CDC Landmark, Waskada and Thatcher) from two different tissues; seedling leaves and developing kernels (five days post anthesis) using NucleoSpin RNA Plant kit (Macherey-Nagel) according to the manufacturer’s instructions. RNA was treated with RNase-free DNase (rDNase) (Macherey-Nagel) and reversed transcribed into cDNA using SuperScript IV Reverse Transcriptase kit (Invitrogen) according to the manufacturer’s instructions and the NB-ARC domain amplified by PCR.

### Reporting summary

Further information on research design is available in the Nature Research Reporting Summary linked to this paper.

## Online content

Any methods, additional references, Nature Research reporting summaries, source data, extended data, supplementary information, acknowledgements, peer review information; details of author contributions and competing interests; and statements of data and code availability are available at 10.1038/s41586-020-2961-x.

## Supplementary information

Supplementary Data Supplementary Figure 1. Original gel source data used for spanning the breakpoint for the 7B/5B translocation.

Reporting Summary

Supplementary Information This file contains Supplementary Notes 1-8.

Supplementary Tables This file contains Supplementary Tables 1-27.

## Data Availability

All sequence reads assemblies have been deposited into the National Center for Biotechnology Information sequence read archive (SRA) (see Supplementary Table 1 for accession numbers). Sequence reads for the RQAs, *T. ponticum*, *A. ventricosa* and *T. timopheevii* have been deposited into the SRA (accession no. PRJNA544491) and ChIP–seq short read-data used for centromere characterization is deposited under accession no. PRJNA625537. All Hi-C data have been deposited in the European Nucleotide Archive (Supplementary Table 1). The RQAs are available for direct user download at https://wheat.ipk-gatersleben.de/. All assemblies and projected annotations are available for comparative analysis at Ensembl Plants (https://plants.ensembl.org/index.html). Comparative analysis viewers are also online for synteny (https://kiranbandi.github.io/10wheatgenomes/, http://10wheatgenomes.plantinformatics.io/) and haplotypes (http://www.crop-haplotypes.com/). Seed stocks of the assembled lines are available at the UK Germplasm Resources Unit (https://www.seedstor.ac.uk/).

## Code availability

Code for custom genome visualizers have been deposited in the public domain for haplotype viewer (https://github.com/Uauy-Lab/pangenome-haplotypes), Pretzel (https://github.com/plantinformatics/pretzel), AccuSyn (https://github.com/jorgenunezsiri/accusyn) and SynVisio (https://github.com/kiranbandi/synvisio). Additional scripts used for ChIP–seq analysis of the centromeres are provided at https://github.com/wheatgenetics/centromere.

## References

[1] The International Wheat Genome Sequencing Consortium (2018). Shifting the limits in wheat research and breeding using a fully annotated reference genome. Science.

[2] Montenegro JD (2017). The pangenome of hexaploid bread wheat. Plant J..

[3] International Wheat Genome Sequencing Consortium (IWGSC) (2014). A chromosome-based draft sequence of the hexaploid bread wheat (*Triticum aestivum*) genome. Science.

[4] He F (2019). Exome sequencing highlights the role of wild-relative introgression in shaping the adaptive landscape of the wheat genome. Nat. Genet..

[5] Pont C (2019). Tracing the ancestry of modern bread wheats. Nat. Genet.

[6] Kassa MT (2016). A saturated SNP linkage map for the orange wheat blossom midge resistance gene Sm1. Theor. Appl. Genet..

[7] Tadesse W (2019). Genetic gains in wheat breeding and its role in feeding the world. Crop Breed. Genet. Genom..

[8] Zhao Q (2018). Pan-genome analysis highlights the extent of genomic variation in cultivated and wild rice. Nat. Genet..

[9] Dubcovsky J, Dvorak J (2007). Genome plasticity a key factor in the success of polyploid wheat under domestication. Science.

[10] Marcussen T (2014). Ancient hybridizations among the ancestral genomes of bread wheat. Science.

[11] Avni R (2017). Wild emmer genome architecture and diversity elucidate wheat evolution and domestication. Science.

[12] Maccaferri M (2019). Durum wheat genome highlights past domestication signatures and future improvement targets. Nat. Genet..

[13] Zimin AV (2017). The first near-complete assembly of the hexaploid bread wheat genome, *Triticum aestivum*. Gigascience.

[14] Winfield MO (2012). Targeted re-sequencing of the allohexaploid wheat exome. Plant Biotechnol. J..

[15] Arora D, Gross T, Brueggeman R (2013). Allele characterization of genes required for rpg4-mediated wheat stem rust resistance identifies *Rpg5* as the R gene. Phytopathology.

[16] Adamski, N. M. et al. A roadmap for gene functional characterisation in crops with large genomes: lessons from polyploid wheat. *eLife* **9**, e55646 (2020). 10.7554/eLife.55646 PMC7093151 32208137

[17] Uauy C (2017). Wheat genomics comes of age. Curr. Opin. Plant Biol..

[18] Mascher M (2017). A chromosome conformation capture ordered sequence of the barley genome. Nature.

[19] Edwards D (2012). Bread matters: a national initiative to profile the genetic diversity of Australian wheat. Plant Biotechnol. J..

[20] Jordan KW (2015). A haplotype map of allohexaploid wheat reveals distinct patterns of selection on homoeologous genomes. Genome Biol..

[21] Paape T (2018). Patterns of polymorphism and selection in the subgenomes of the allopolyploid *Arabidopsis kamchatica*. Nat. Commun..

[22] Paape T (2016). Conserved but attenuated parental gene expression in allopolyploids: Constitutive zinc hyperaccumulation in the allotetraploid *Arabidopsis kamchatica*. Mol. Biol. Evol..

[23] Melonek J, Stone JD, Small I (2016). Evolutionary plasticity of restorer-of-fertility-like proteins in rice. Sci. Rep..

[24] Bernhard T, Koch M, Snowdon RJ, Friedt W, Wittkop B (2019). Undesired fertility restoration in msm1 barley associates with two mTERF genes. Theor. Appl. Genet..

[25] Whitford R (2013). Hybrid breeding in wheat: technologies to improve hybrid wheat seed production. J. Exp. Bot..

[26] Keller B, Wicker T, Krattinger SG (2018). Advances in wheat and pathogen genomics: Implications for disease control. Annu. Rev. Phytopathol..

[27] Steuernagel B (2016). Rapid cloning of disease-resistance genes in plants using mutagenesis and sequence capture. Nat. Biotechnol..

[28] Bariana HS (2001). Mapping of durable adult plant and seedling resistances to stripe rust and stem rust diseases in wheat. Aust. J. Agric. Res..

[29] Chemayek B (2017). Tight repulsion linkage between *Sr36* and *Sr39* was revealed by genetic, cytogenetic and molecular analyses. Theor. Appl. Genet..

[30] Cruz CD (2016). The 2NS translocation from *Aegilops ventricosa* confers resistance to the *Triticum* pathotype of *Magnaporthe oryzae*. Crop Sci..

[31] Helguera M (2003). PCR assays for the Lr37-Yr17-*Sr38* cluster of rust resistance genes and their use to develop isogenic hard red spring wheat lines. Crop Sci..

[32] Li Y, Wei K (2020). Comparative functional genomics analysis of cytochrome P450 gene superfamily in wheat and maize. BMC Plant Biol..

[33] Gunupuru LR (2018). A wheat cytochrome P450 enhances both resistance to deoxynivalenol and grain yield. PLoS ONE.

[34] Li B (2013). Wheat centromeric retrotransposons: the new ones take a major role in centromeric structure. Plant J..

[35] Gent JI, Wang K, Jiang J, Dawe RK (2015). Stable patterns of CENH3 occupancy through maize lineages containing genetically similar centromeres. Genetics.

[36] Koo DH, Sehgal SK, Friebe B, Gill BS (2015). Structure and stability of telocentric chromosomes in wheat. PLoS ONE.

[37] Schneider KL, Xie Z, Wolfgruber TK, Presting GG (2016). Inbreeding drives maize centromere evolution. Proc. Natl Acad. Sci. USA.

[38] Saxena RK, Edwards D, Varshney RK (2014). Structural variations in plant genomes. Brief. Funct. Genomics.

[39] Harewood L (2017). Hi-C as a tool for precise detection and characterisation of chromosomal rearrangements and copy number variation in human tumours. Genome Biol..

[40] Himmelbach A (2018). Discovery of multi-megabase polymorphic inversions by chromosome conformation capture sequencing in large-genome plant species. Plant J..

[41] Fradgley N (2019). A large-scale pedigree resource of wheat reveals evidence for adaptation and selection by breeders. PLoS Biol..

[42] Martín AC, Rey MD, Shaw P, Moore G (2017). Dual effect of the wheat Ph1 locus on chromosome synapsis and crossover. Chromosoma.

[43] Bevan MW (2017). Genomic innovation for crop improvement. Nature.

[44] Luján Basile SM (2019). Haplotype block analysis of an Argentinean hexaploid wheat collection and GWAS for yield components and adaptation. BMC Plant Biol..

[45] Fox SL (2010). Unity hard red spring wheat. Can. J. Plant Sci..

[46] Hanks SK, Quinn AM, Hunter T (1988). The protein kinase family: conserved features and deduced phylogeny of the catalytic domains. Science.

[47] Brueggeman R (2008). The stem rust resistance gene *Rpg5* encodes a protein with nucleotide-binding-site, leucine-rich, and protein kinase domains. Proc. Natl Acad. Sci. USA.

[48] Faris JD (2010). A unique wheat disease resistance-like gene governs effector-triggered susceptibility to necrotrophic pathogens. Proc. Natl Acad. Sci. USA.

[49] Luo MC (2017). Genome sequence of the progenitor of the wheat D genome *Aegilops tauschii*. Nature.

[50] Borrill P, Harrington SA, Uauy C (2019). Applying the latest advances in genomics and phenomics for trait discovery in polyploid wheat. Plant J..

[51] Dvorak J, Mcguire PE, Cassidy B (1988). Apparent sources of the A genomes of wheats inferred from polymorphism in abundance and restriction fragment length of repeated nucleotide-sequences. Genome.

[52] Watson-Haigh NS, Suchecki R, Kalashyan E, Garcia M, Baumann U (2018). DAWN: a resource for yielding insights into the diversity among wheat genomes. BMC Genomics.

[53] Kent WJ (2002). BLAT—the BLAST-like alignment tool. Genome Res..

[54] Slater GS, Birney E (2005). Automated generation of heuristics for biological sequence comparison. BMC Bioinformatics.

[55] Tatusov RL, Galperin MY, Natale DA, Koonin EV (2000). The COG database: a tool for genome-scale analysis of protein functions and evolution. Nucleic Acids Res..

[56] Thornton K (2003). Libsequence: a C++ class library for evolutionary genetic analysis. Bioinformatics.

[57] Cheng S (2016). Redefining the structural motifs that determine RNA binding and RNA editing by pentatricopeptide repeat proteins in land plants. Plant J..

[58] Steuernagel, B. et al. Physical and transcriptional organisation of the bread wheat intracellular immune receptor repertoire. Preprint at 10.1101/339424 (2018).

[59] Steuernagel B (2020). The NLR-Annotator tool enables annotation of the intracellular immune receptor repertoire. Plant Physiol..

[60] Spannagl M (2016). PGSB PlantsDB: updates to the database framework for comparative plant genome research. Nucleic Acids Res..

[61] Benson G (1999). Tandem repeats finder: a program to analyze DNA sequences. Nucleic Acids Res..

[62] Nagaki K (2003). Chromatin immunoprecipitation reveals that the 180-bp satellite repeat is the key functional DNA element of *Arabidopsis thaliana* centromeres. Genetics.

[63] Guo X (2016). De novo centromere formation and centromeric sequence expansion in wheat and its wide hybrids. PLoS Genet..

[64] Kim D, Paggi JM, Park C, Bennett C, Salzberg SL (2019). Graph-based genome alignment and genotyping with HISAT2 and HISAT-genotype. Nat. Biotechnol..

[65] Wicker T (2018). Impact of transposable elements on genome structure and evolution in bread wheat. Genome Biol..

[66] Tang Z, Yang Z, Fu S (2014). Oligonucleotides replacing the roles of repetitive sequences pAs1, pSc119.2, pTa-535, pTa71, CCS1, and pAWRC.1 for FISH analysis. J. Appl. Genet..

[67] Zhao L (2016). Cytological identification of an *Aegilops variabilis* chromosome carrying stripe rust resistance in wheat. Breed. Sci..

[68] Komuro S, Endo R, Shikata K, Kato A (2013). Genomic and chromosomal distribution patterns of various repeated DNA sequences in wheat revealed by a fluorescence in situ hybridization procedure. Genome.

[69] Li H (2018). Minimap2: pairwise alignment for nucleotide sequences. Bioinformatics.

[70] Kubaláková M, Vrána J, Cíhalíková J, Simková H, Doležel J (2002). Flow karyotyping and chromosome sorting in bread wheat (*Triticum aestivum* L.). Theor. Appl. Genet..

[71] Brinton, J. et al. A haplotype-led approach to increase the precision of wheat breeding. *Commun. Biol.* https://doi.org/10.1038/s42003-020-01413-2 (2020). 10.1038/s42003-020-01413-2 PMC7689427 33239669

[72] Thomas J (2005). Chromosome location and markers of Sm1: a gene of wheat that conditions antibiotic resistance to orange wheat blossom midge. Mol. Breed..

[73] Lamb RJ (2000). Resistance to *Sitodiplosis mosellana* (Diptera: Cecidomyiidae) in spring wheat (Gramineae). Can. Entomol..

[74] la Cour T (2004). Analysis and prediction of leucine-rich nuclear export signals. Protein Eng. Des. Sel..

