# Supplementary material for: Multiple wheat genomes reveal global variation in modern breeding
Source: Nature. 2020 Nov 25;588(7837):277–83. doi: 10.1038/s41586-020-2961-x (PMC7759465; doi:10.1038/s41586-020-2961-x)
Supplement: Supplementary file 1 — Supplementary Figure 1. Original gel source data used for spanning the breakpoint for the 7B/5B translocation. [file 41586_2020_2961_MOESM1_ESM.pdf]

natureresearch

https://doi.org/10.1038/s41586-020-2961-x

Supplementary information

Multiple wheat genomes reveal global variation in modern breeding

In the format provided by the authors and unedited

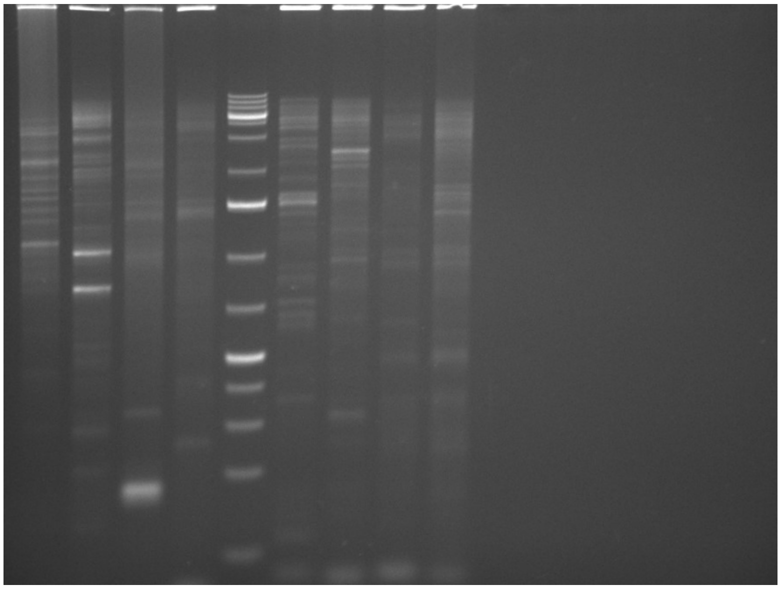

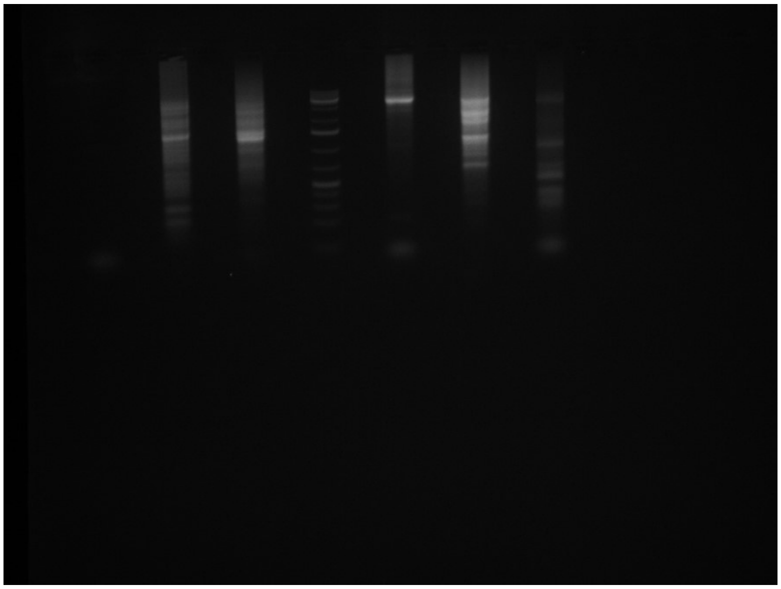

Supplementary Figure 1 | Original gel source data used for spanning the breakpoint for the 7B/5B translocation. Images were cropped and used in the construction of Extended Data Figure 9. A DNA ladder is included in the middle of each image for approximating the size of the amplicons.
