# Supplementary material for: Multiple wheat genomes reveal global variation in modern breeding
Source: Nature. 2020 Nov 25;588(7837):277–83. doi: 10.1038/s41586-020-2961-x (PMC7759465; doi:10.1038/s41586-020-2961-x)
Supplement: Supplementary file 2 — Reporting Summary [file 41586_2020_2961_MOESM2_ESM.pdf]

nature research

Corresponding author(s): Curtis Pozniak

Last updated by author(s): Aug 11, 2020

# Reporting Summary

Nature Research wishes to improve the reproducibility of the work that we publish. This form provides structure for consistency and transparency in reporting. For further information on Nature Research policies, see Authors & Referees and the Editorial Policy Checklist.

## Statistics

For all statistical analyses, confirm that the following items are present in the figure legend, table legend, main text, or Methods section.

| n/a | Confirmed | |
|---|---|---|
| ☐ | ☒ | The exact sample size (*n*) for each experimental group/condition, given as a discrete number and unit of measurement |
| ☐ | ☒ | A statement on whether measurements were taken from distinct samples or whether the same sample was measured repeatedly |
| ☐ | ☒ | The statistical test(s) used AND whether they are one- or two-sided<br>*Only common tests should be described solely by name; describe more complex techniques in the Methods section.* |
| ☒ | ☐ | A description of all covariates tested |
| ☐ | ☒ | A description of any assumptions or corrections, such as tests of normality and adjustment for multiple comparisons |
| ☐ | ☒ | A full description of the statistical parameters including central tendency (e.g. means) or other basic estimates (e.g. regression coefficient) AND variation (e.g. standard deviation) or associated estimates of uncertainty (e.g. confidence intervals) |
| ☐ | ☒ | For null hypothesis testing, the test statistic (e.g. *F*, *t*, *r*) with confidence intervals, effect sizes, degrees of freedom and *P* value noted<br>*Give P values as exact values whenever suitable.* |
| ☐ | ☒ | For Bayesian analysis, information on the choice of priors and Markov chain Monte Carlo settings |
| ☐ | ☒ | For hierarchical and complex designs, identification of the appropriate level for tests and full reporting of outcomes |
| ☒ | ☐ | Estimates of effect sizes (e.g. Cohen's *d*, Pearson's *r*), indicating how they were calculated |

*Our web collection on statistics for biologists contains articles on many of the points above.*

## Software and code

Policy information about availability of computer code

| | |
|---|---|
| Data collection | No software was used to collect data for this study. |
| Data analysis | A multitude of software and databases were used in this study, all of which have been listed, cited, or provided. These include: DeNovoMAGIC v3.0, W2RAP (no versions, https://github.com/bioinfologics/w2rap), LongRanger v2.1.6, GATK v3.8, R v3.6.1 and v3.0.2, BLAT v3.5, BLAST v2.8 , MUSCLE v3.8, libsequence v1.8.3, EMBOSS v6.6.0, HMMER 3.1b2, PFAM v32.0, NLR-Annotator (no version, https://github.com/steuernb/NLR-Annotator), Vmatch v2.3.0, TandemRepeatFinder v4.07b, LTRharvest genometools-1.5.9, HMMER v3.0, MUMmer v3.23 (haplotype database) and v4 (all other analyses), HISAT v2.1.0, SNPrelate v3.11, BBTools/BBMap v38, ImageJ v1.51n, minimap2 v2.13, FGENESH v2.6, NCBI Conserved Domain Search tool (no version, https://www.ncbi.nlm.nih.gov/Structure/cdd/wrpsb), PROSITE release 2020_01, TMpred v25, STAR v2.6.0b., AUGUSTUS v3.2.3., GMAP v2017-06-20, EvidenceModeler v1.1.1, AHRD v1.6, MCScanX v2.0, samtools v1.10, BEDtools v2.29, and custom data scripts (https://github.com/Uauy-Lab/pangenome-haplotypes; http://people.beocat.ksu.edu/~jpoland/centromeres/). |

For manuscripts utilizing custom algorithms or software that are central to the research but not yet described in published literature, software must be made available to editors/reviewers. We strongly encourage code deposition in a community repository (e.g. GitHub). See the Nature Research guidelines for submitting code & software for further information.

## Data

Policy information about availability of data

All manuscripts must include a data availability statement. This statement should provide the following information, where applicable:

- Accession codes, unique identifiers, or web links for publicly available datasets
- A list of figures that have associated raw data
- A description of any restrictions on data availability

All sequence reads have been deposited into the National Center for Biotechnology Information sequence read archive (SRA) (see Supplementary Table 1 for accession numbers). Sequence reads for the RQAs, Th. ponticum, Ae. ventricosa and T. timopheevii have been deposited into the SRA (no. PRJNA544491) and ChIP-

seq short read-data used for centromere characterization is deposited as PRJNA625537. All Hi-C data has been deposited in the European Nucleotide Archive (Supplementary Table 1). The RQAs and projected annotations are available for direct user download at https://wheat.ipk-gatersleben.de/. All RQA assemblies have also been deposited at EBI with the following accession numbers: GCA_903993795; GCA_903993985; GCA_903993975; GCA_903994175; GCA_903994195; GCA_904066035; GCA_903994155; GCA_903994165; GCA_903994185; GCA_903995565. These data will be syncrhonized across multiple platforms including NCBI and at Ensembl Plants (https://plants.ensembl.org/index.html). Comparative analysis viewers are also online for synteny (https://kiranbandi.github.io/10wheatgenomes/; http://10wheatgenomes.plantinformatics.io/) and haplotypes (http://www.crop-haplotypes.com/). Seed stocks of the assembled lines are available at the UK Germplasm Resources Unit (https://www.seedstor.ac.uk/).

# Field-specific reporting

Please select the one below that is the best fit for your research. If you are not sure, read the appropriate sections before making your selection.

☒ Life sciences ☐ Behavioural & social sciences ☐ Ecological, evolutionary & environmental sciences

For a reference copy of the document with all sections, see nature.com/documents/nr-reporting-summary-flat.pdf

# Life sciences study design

All studies must disclose on these points even when the disclosure is negative.

| | |
|---|---|
| Sample size | No statistical methods were used to establish sample size. The samples that were sequenced were selected to represent modern breeding material from different continents that had known differences in pedigree and were known to carry different genes/traits/chromosomal segments of interest. |
| Data exclusions | All sequencing data generated was used in the genome assembly and analyses. Whenever possible, all data was included in the supporting analyses. Data exclusion applies only to some of the subsequent supporting analysis, which was pre-established based on limitations in the data. For example, we excluded the scaffolded assemblies from some analyses because the analyses required chromosome pseudomolecules. We performed diversity analysis both with the spelt genome but also excluding the spelt genome because it is a different species and is much more diverged and biased the results. |
| Replication | In all analyses that support the genome assemblies, the number of replicates or iterations are indicated in materials and methods or supplemental tables. In each case, all replications were successful and were used. The genome assemblies themselves were validated using multiple methods (i.e. BUSCO, genetic maps, HiC, 10x Genomics, cytology, and comparions to Chinese Spring). The CDC Landmark assembly was further validated using Oxford Nanopore long read sequencing. This helped validate the other approaches. |
| Randomization | Randomization does not directly apply to the genome sequencing and assembly; however it applies to some of the supporting analyses. In these cases, the group design and data seeding for computational analysis are described in the materials and methods and adhere to widely accepted standards. For example, analysis of NLRs (Fig. 1c), 1 million random permutations were used. For the field experiments established for phenotyping of Sm1, all samples were replicated and randomized using appropriate experimental designs. |
| Blinding | Blinding does not apply to this study, as the study focuses on genome sequencing. This study focuses on plants genomics and the results of the study are not impacted by the concealment of treatment, data, or groups. |

# Reporting for specific materials, systems and methods

We require information from authors about some types of materials, experimental systems and methods used in many studies. Here, indicate whether each material, system or method listed is relevant to your study. If you are not sure if a list item applies to your research, read the appropriate section before selecting a response.

## Materials & experimental systems

| n/a | Involved in the study | |
|---|---|---|
| ☐ | ☒ | Antibodies |
| ☒ | ☐ | Eukaryotic cell lines |
| ☒ | ☐ | Palaeontology |
| ☒ | ☐ | Animals and other organisms |
| ☒ | ☐ | Human research participants |
| ☒ | ☐ | Clinical data |

## Methods

| n/a | Involved in the study | |
|---|---|---|
| ☐ | ☒ | ChIP-seq |
| ☒ | ☐ | Flow cytometry |
| ☒ | ☐ | MRI-based neuroimaging |

## Antibodies

| | |
|---|---|
| Antibodies used | Chromatin immunoprecipitation (ChIP) was performed ausing wheat CenH3 antibody (Koo et al., 2015). A antigen with the peptide sequence 'RTKHPAVRKTKALPKK' corresponding to the N-terminus of wheat CENH3 was used to produce antibody utilizing the custom-antibody production facility provided by the Thermo Fisher Scientific, Illinois, USA (abs@thermofisher.com). A 0.396 mg of the antibody pellet was dissolved in 2 ml of PBS buffer, pH 7.4 resulting in 198 ng/uL of the working concentration. |
| Validation | In the manuscript, we validate the antibody according to a previous study of Chinese Spring (Koo et al., 2015) and achieved near |

identical results (Supplementary Table 12). Additional controls were used in the study where the antibody was substituted with rabbit serum, which serves as nonspecific binding control in chromatin immunoprecipitation assay.

# ChIP-seq

## Data deposition

☒ Confirm that both raw and final processed data have been deposited in a public database such as GEO.

☒ Confirm that you have deposited or provided access to graph files (e.g. BED files) for the called peaks.

| | |
|---|---|
| Data access links<br>*May remain private before publication.* | The data for the project has been deposited at NCBI: PRJNA625537 and analysis files are available for download: http://people.beocat.ksu.edu/~jpoland/centromeres/ |
| Files in database submission | BED files, delta files (MUMmer), data analysis scripts |
| Genome browser session<br>(e.g. UCSC) | Data for visualization is available at http://people.beocat.ksu.edu/~jpoland/centromeres/ |

## Methodology

| | |
|---|---|
| Replicates | NA. Samples were obtained from 2-week-old seedlings. |
| Sequencing depth | Paired-end reads were generated at varying levels of read depth, data was deposited at NCBI (PRJNA625537). |
| Antibodies | Wheat CenH3 antibody - see: Koo DH, Sehgal SK, Friebe B, Gill BS (2015) Structure and stability of telocentric chromosomes in wheat. PLoS One 10: e0137747. |
| Peak calling parameters | Reads mapped per 100kb bin were counted for each sample using BEDtools and output as a bed file. Scripts for data analysis are provided at http://people.beocat.ksu.edu/~jpoland/centromeres/. Unlike studies involving transcription factors, CENH3 ChIP-seq provides clear distinct peaks that are ~100 fold greater than background. |
| Data quality | SAM output files from HISAT2 were converted to BAM, sorted and filtered for minimum alignment quality of 30 using SAMtools. |
| Software | Reads for each sample were aligned to each of the respective genome assemblies using HISAT2.Reads mapped per 100kb bin were counted for each sample using BEDtools and output as a bed file. Scripts for data analysis are provided at http://people.beocat.ksu.edu/~jpoland/centromeres/. |
