# Supplementary material for: Multiple wheat genomes reveal global variation in modern breeding
Source: Nature. 2020 Nov 25;588(7837):277–83. doi: 10.1038/s41586-020-2961-x (PMC7759465; doi:10.1038/s41586-020-2961-x)
Supplement: Supplementary file 3 — This file contains Supplementary Notes 1-8. [file 41586_2020_2961_MOESM3_ESM.pdf]

SUPPLEMENTARY NOTE 1

ASSEMBLY METRICS

We sequenced and assembled 15 wheat genomes. **Supplementary Table 1** provides passport information for these lines. Seed stocks of the assembled lines are available at the UK Germplasm Resources Unit (https://www.seedstor.ac.uk/).

RQA Assemblies

Paired-end, mate-pair, 10X Genomics Chromium linked reads, and Hi-C data were generated and assembled using the DenovoMagic3.0 pipeline as described previously for other cereals (See Methods). The assemblies were 14.2-14.8 Gb in length and consisted of 487,203-1,058,304 contigs and 99,465-701,145 scaffolds (**Supplementary Table 2**). POPSEQ (ftp://ftp.ipk-gatersleben.de/barley-popseq) and Hi-C was then used to break chimeric scaffolds (corrected assembly), and then order and orient the scaffolds into chromosome pseudomolecules that were ~14 Gb in length, as was done previously for Chinese Spring RefSeq v1.0 (**Supplementary Table 2**). The assemblies contained ~98% of the BUSCO gene complement (**Supplementary Table 2**) and were highly collinear to the assembly of Chinese Spring (**Extended Data Fig. 1**), thereby validating their quality and completeness.

Scaffolded Assemblies

Paired-end and mate-pair libraries were constructed and sequenced at the Earlham Institute by the Genomics Pipelines Group. A total of 2 µg of DNA was sheared targeting 1 kb fragments on a Covaris- S2, size selected on a Sage Science Blue Pippin 1.5% cassette to remove DNA molecules <600 bp, and amplification-free, paired-end libraries were constructed using the Kapa Biosciences Hyper Prep Kit. Mate-pair libraries with insert sizes >7 kb were constructed from 9 µg of DNA based on the Illumina Nextera mate-pair kits. Sequencing was performed on an Illumina HiSeq 2500 instrument, which generated 250 bp reads at a read depth of ~45 for the pair-end and ~25 for the mate-paired sequencing libraries.

A read depth between 44 and 51 was generated per line. Contig construction was performed using the w2rap-contigger using k=200. Two mate-pair libraries were produced for each line except Weebill 1, where five libraries were used. Mate-pairs were processed, filtered, and used to scaffold contigs as described in the W2RAP pipeline (https://github.com/bioinfologics/w2rap). Scaffolds >500 bp were removed from the final assemblies (**Supplementary Table 3**). The k-mer Analysis Toolkit (https://github.com/TGAC/KAT) was used to validate the scaffolds by generating a k-mer histogram from the matrix of k-mers shared between the paired-end reads and the scaffolds.

SUPPLEMENTARY NOTE 2

OXFORD NANOPORE SEQUENCING OF CDC LANDMARK

The order and orientation of the scaffolds in each of the pseudomolecules for the CDC Landmark RQA were determined using Hi-C**,** as was performed previously for the published genomes of Chinese Spring, Svevo (durum wheat), and Zavitan (wild emmer wheat). This method was used for pseudomolecule assembly (**Supplementary Note 1**) and for calling large scale (>1 Mb) inversions (**Supplementary Table 13; Extended Data Fig. 8**) due to the large distances (several Mb) that can be captured by Hi-C contact maps and high base-level accuracy of Illumina sequencing. To validate this method, we used long reads generated from the Oxford Nanopore Technology (ONT) sequencing platform to identify reads spanning the gaps between the neighbouring scaffolds, thereby confirming the scaffold placement by Hi-C. We performed long read sequencing of CDC Landmark; high molecular weight DNA was isolated from wheat seedlings and size selected as was performed for the 10x Genomics Chromium sequencing. Libraries for whole-genome ONT sequencing were prepared following the standard ligation protocol (LSK109) and sequenced on a GridION instrument using standard parameters and R9 flow cells. The sequencing generated ~72 million reads with a read N50 of ~15 kb, which is equivalent to a ~30 fold sequencing read-depth of CDC Landmark. The sequence reads were aligned to the RQA of CDC Landmark and Chinese Spring using Minimap2 v.2.1.0. Next, we extracted the read IDs for only those ONT long reads that mapped within a 5 kb region at both ends of each scaffold. We then compared read IDs between scaffold ends to identify those reads that mapped to both neighbouring scaffolds placed in the pseudomolecules, as well as to the ends of scaffolds from other locations in the RQA. The results demonstrate that adjacent scaffolds were 94 times more likely to share read IDs, thus corroborating the scaffold placement by Hi-C. The overlap in read IDs between scaffold junctions was used to construct a scaffold-scaffold contact map across the ordered and oriented scaffolds in the CDC Landmark pseudomolecules (**Extended Data Fig. 2a**).

We also manually inspected the breakpoints of structural variants that we identified from aligning the Hi-C data from CDC Landmark to the Chinese Spring RQA (**Supplementary Table 13**). For example, chromosome alignments and Hi-C predicted inversions on chromosomes 2A, 3B, and 3D when comparing CDC Landmark to Chinese Spring (**Extended Data Fig. 2b**). Manual inspection of the ONT long reads mapped to the assembly of Chinese Spring identified reads that whose alignment were interrupted at the breakpoints of this inversion event, which mapped to the (+) and (-) strand of the assembly on either side of the breakpoint, thereby supporting the inversion events between assemblies (**Extended Data Fig. 2b**).

Furthermore, we validated the gene structure of *Sm1* using the ONT read mappings, given that CDC Landmark is a carrier of that gene (**Fig. 3**). Inspection of the region containing the candidate gene for *Sm1* on chromosome 2B of CDC Landmark also confirmed a uniform distribution of read depth, as well as many long reads that were able to completely span the candidate gene (**Extended Data Fig. 10b**). However, alignment of CDC Landmark long reads to corresponding region in the Chinese Spring assembly that only contains a partial gene sequence revealed irregular patterns of read depth, as well as an abundance of point mutations, thereby supporting an alternate haplotype (**Extended Data Fig. 10b**).

In summary, we used the established RQA assembly pipeline that was used previously used for Chinese Spring, Svevo, and Zavitan, to generate new RQA for wheat lines of interest for global breeding programs. Using ONT long reads, we demonstrate that this assembly approach is able to properly order and orient scaffolds in pseudomolecules and identify structural variation between assemblies. We also demonstrate that the RQA assembly pipeline was able to properly assemble the gene candidate for *Sm1*.

87

SUPPLEMENTARY NOTE 3

VARIATION IN GENES

Gene Projections

We used the previously published RefSeq v1.0 high-confidence gene models for Chinese Spring to assess the gene content in each assembly (See Online Methods). Projections were filtered using several iterations to ensure the best projected match was used. The first iteration required a contiguous ORF and informant coverage ≥ 99% on an orthologous chromosome, the second removed the chromosome orthology requirement, the third iteration relaxed the informant coverage to equal or greater than 90%, and the fourth step allowed disrupted/non-contiguous ORFs restricting informant coverage to ≥ 95%. For the fifth iteration, we projected all remaining matches with a contiguous ORF comprising a start and stop codon, an informant coverage ≥ 5% and a genome-wide mapping frequency <50 copies. In a last step, we projected all matches from informants missed in previous iterations if their alignment coverage exceeded 80%. To provide uniformity in the data, Informants were also projected onto the Chinese Spring assembly. This involved first masking existing gene coding regions and iteratively adding genes with the first iteration including high coverage (≥ 99%) matches, then adding matches with a contiguous ORF and a mapping frequency ≤ 10, and then genes with up to 50 matches per genome.

We identified tandem gene clusters and OGs (orthologous groups; See Online Methods) to quantify unique genes and those displaying PAV and CNV patterns. Next, we surveyed CDS similarities between all pairwise combinations of cultivars using BLATn v3.5. Next, we generated an undirected graph with gene accessions as nodes and edges representing matches between two genes and identified OGs and tandem clusters as subgraphs/connected components restricted to particular edge attributes. OGs were restricted to RBH relations, while tandem clusters required an e-value ≤ 1^{-30}, an alignment coverage of at least 50% for both, the query and database accession, and a maximal genomic distance of nine unrelated genes between both accessions. PAV genes per line were determined from the orthologous groups limited to one-to-one relations and comprising 2-10 cultivars. An absence variant was counted for each group and for each line if a group missed an orthologous gene of the respective line. Hence, the reported PAV count (**Supplementary Table 5**) is the sum of the number of genes per line that are missing in the union of all orthologous groups. In contrast, computation of CNV genes per line has been restricted to orthotandem clusters (i.e. the orthologous groups above complemented by tandem duplications) that comprised at least one gene of the respective line. Thereby, CNV counts ignore any cluster which misses the respective line and rather describe the expansion of copies. Expansion is defined as the number of gene copies per line which are redundant to the minimum copy number that each of the lines contribute to the cluster. We limit our analysis to the one-to-one groups and their extension to orthotandem clusters to ensure high accuracy. It should be emphasized that the group 'other' comprises genes that are either singletons in a line or participate in complex relationships where at least one line contributes more than one copy to the orthologous group. Albeit in the latter cases there are certainly copy number variations, ambiguous orthologous assignments will highly increase the errors in PAV counts. Overall, it should be stressed that the PAV counts are likely inflated by the orthologous groups with very low line counts (2-7) which trigger in each case for a large number of lines an absence count. From our experience, many of these groups represent genes with questionable or spurious functionality.

Genetic Diversity of A, B and D Homeologs Suggests Increased Targets of Selection

To estimate the pattern of genome-wide polymorphisms of wheat cultivars, we analysed the coding sequences by using gene alignments of the *de novo* genome assemblies. To derive codon-based

alignments, we aligned amino acid sequences of one OG (excluding gene models with disrupted ORFs; see above) using MUSCLE v3.8 with default parameters and then back translated aligned codons to nucleotide alignments. We also only used alignments where each homeolog was found in all RQA used in the analysis. Average pairwise nucleotide diversity (π) and θ_W was estimated and their units are reported as per base pair. By taking the mean values of π for each subgenome, we found that the genetic diversity of the A and B subgenomes (0.0012 and 0.0021, respectively) are higher than that of the D subgenome (0.0004) (**Supplementary Table 6**). The values were highly similar in both datasets with and without PI190962 (spelt wheat), although lower Tajima's *D* in the dataset with PI190962 indicates an excess of rare variants in PI190962. Because PI190962 may have a complex history involving hybridization with wild species, we focused only on the analysis of just the bread wheat lines. The peak π distribution for the three subgenomes was similar to previous studies, which indicates that the RQA were able to capture similar patterns of diversity as larger diversity panels representing global breeding programs.

Synonymous (or silent site) nucleotide diversity (π_sil) was approximately double the total site π for each subgenome (π_sil = 0.0024, 0.0046, and 0.0009 for A, B, and D subgenomes, respectively (**Supplementary Table 7**). The level of polymorphism of the A and B subgenomes is similar to the natural allopolyploid *Arabidopsis kamchatica* (0.0014-0.0015 in total sites, 0.0044-0.0049 in synonymous sites), suggesting that bread wheat retained considerable global variation comparable to wild species, despite domestication and polyploidization. The patterns are consistent with a recent report showing higher variation in the A and B subgenomes than in the D subgenome. The mean and median of Tajima's *D* of the D subgenome is lower than the A and B subgenomes (**Extended Data Fig. 3 a,b**), indicating an excess of rare variants in the D subgenome.

Next, we tested whether homeologous pairs experienced similar or different evolutionary trajectories in a genome-wide manner. In wheat, it is known that the level of polymorphism along each chromosome is positively correlated with the distance from centromere, which should result in a positive correlation of π_sil between homeologs. Yet, the correlations among homeologs were low (0.11-0.29) (**Supplementary Table 8**), suggesting different evolutionary trajectories of homeologs. More importantly, the correlation of the neutrality statistic, Tajima's *D*, was very low between pairs of homelogs (*r* = 0.02-0.06) among the three subgenomes, which again supports that homeologous copies experienced different selective pressure. These results are in line with our current understanding that selective sweeps rarely occur in homeologous regions in bread wheat.

SUPPLEMENTARY NOTE 4

SEQUENCE DIVERSITY OF *RF* LOCI IN WHEAT

Despite the promise of higher yield and better stress resistance of hybrid varieties compared to conventional lines, hybrid breeding in wheat remains underexploited. An efficient pollination control-system that would be easy to apply on a commercial scale is missing. The application of cytoplasmic male sterility (CMS) and restorer-of-fertility (*Rf*) genes have been successful in other plant species such as rice and maize, but is difficult to use in wheat due to poor effectiveness of the known wheat *Rf* genes. In most flowering plants, the majority of *Rf* genes belong to the pentatricopeptide repeat (PPR) family encoding mitochondrial sequence-specific RNA binding proteins. The PPR family can be split into two classes based on their motif architecture, and this distinction correlates with function: P-class PPR proteins are implicated in a wide range of RNA processing activities whilst PLS-class PPR proteins are almost exclusively implicated in RNA editing. *Rf* genes are found within a subclade (Rf-like, or RFL genes) of the P-class.

Discovery of RFL-mTERF Clade and its Expansion in Wheat

Members of another family of sequence-specific organellar nucleic acid binding proteins, the mitochondrial transcription termination factor (mTERF) family, may also act in fertility restoration in plants. The wheat genome contains ~400 mTERF sequences of which more than 300 are found in clusters overlapping with RFL gene clusters (**Supplementary Table 9**). For comparison, in *Arabidopsis*, there are 35 mTERF genes, of which 25 are distributed evenly across the genome and are implicated in RNA-associated processes in chloroplasts and mitochondria. Based on sequence similarity, these 25 genes are putatively orthologous to the ~28-30 scattered mTERF sequences found on each of the three subgenomes in wheat (**Supplementary Table 9**). A single cluster of ~10 mTERF genes of unknown function from *Arabidopsis* may correspond to the huge clusters of mTERF genes found in wheat and other cereals including rye and barley. The discovery of clustered mTERF sequences that share genome locations and the same patterns of evolution as *RFL*-type PPR genes is a strong indication that they play a major and hitherto unappreciated role in fertility restoration in cereals.

SUPPLEMENTARY NOTE 5

AEGILOPS VENTRICOSA $2N^{v}S$ SEGMENT FROM *VPM-1*

Based on pedigree and marker analyses, it was previously known that the *Aegilops ventricosa* $2N^{v}S$ segment from the *Vpm-1* introgression is present in several wheat cultivars including Jagger. We generated a RQA of Jagger (**Supplementary Note 1**) and delineated the $2N^{v}S$ segment to be ~33 Mb based on patterns of RLC Angela elements (**Extended Data Fig. 5**) and chromosome alignments to Chinese Spring chromosome 2A (**Extended Data Fig. 6a**), which was further corroborated using genomic *in situ* hybridization (GISH) technology with NN genome probes (results not shown). The pattern in *RLC Angela* elements and chromosome alignments also revealed that in addition to Jagger, CDC Stanley, SY Mattis, and Mace are also carriers of the same introgression. We also observed a region within the spelt genome that was dissimilar to both chromosome 2A and the $2N^{v}S$ introgression, suggesting that alternative haplotypes may exist in this region (**Extended Data Fig. 6a, arrow**). An alternative haplotype is also supported by the analysis of unique RLC Angela elements in that region in the spelt genome assembly (**Extended Data Fig. 5**).

We collected various tissue types from Jagger after being grown until different growth stages and conducted RNA sequencing. Sequencing data was deposited to NCBI SRA (**Supplementary Table 17**). We annotated the Jagger $2N^{v}S$ segment using a combination of *ab initio* predictions and RNA-seq evidence. First, we mapped the ~3 billion RNA-seq reads against the Jagger genome assembly using STAR v2.6.0b. Intron and exon structures were predicted based on RNA-seq alignments, which were combined with *ab initio* gene prediction by AUGUSTUS v3.2.3. Second, we *de novo* assembled RNA-seq transcripts using Trinity and mapped these back to the genome using GMAP (v 2017-06-20). EvidenceModeler v1.1.1 was used to combine the *ab initio* predictions and mapped transcripts, resulting in a set of candidate gene models. We further differentiated the candidates derived from EvidenceModeler into classes of bona-fide genes, non-coding transcripts, pseudogenes and TEs. We then applied a confidence classification protocol similar to the one applied to Chinese Spring RefSeqv1.0, based on coverage and hits in the PTREP, UniPoa, and UniMag databases. The result was a set of high-confidence (HC) genes (**Supplementary Table 18**). Finally, we assigned a functional annotation and human readable description to the HC genes using AHRD v1.6 (https://github.com/groupschoof/AHRD) (**Supplementary Table 18**). Analysis revealed several groups of related genes, including genes encoding disease resistance proteins (i.e. NB-ARC and NLR genes), cytochrome P450s, transporters, chalcone synthases, glycosyltransferase, sulfotransferase, and proteases. We identified orthologous genes between Jagger $2N^{v}S$ and Chinese Spring chromosomes 2A, 2B, 2D based on RBH. Orthologous genes and genomic distributions were visualized using Circos (**Extended Data Fig. 6b**).

We used genotyping-by-sequencing (GBS) to predict the presence of $2N^{v}S$ in three datasets: (1) Kansas State University winter wheat, (2) USDA Regional Performance Nursery, and (3) International Maize and Wheat Improvement Center (CIMMYT) spring wheat (**Supplementary Table 19**). The prediction was based on relative count of wheat and alien specific GBS tag numbers. The frequency of $2N^{v}S$ carriers increased in the three datasets (**Extended Data Fig. 6c**), reaching ~80% for CIMMYT and Kansas State University breeding lines in recent years. These results suggest that $2N^{v}S$ carrier varieties were collectively grown in tens of millions of hectares throughout the world. We also studied the relationship between $2N^{v}S$ presence and wheat grain yield, our results suggest that the $2N^{v}S$ segment is providing a yield benefit in majority of the years studied (**Extended Data Fig. 6d; Supplementary Table 20**). We analysed the data by fitting a mixed linear model with the presence of $2N^{v}S$ as having fixed effects and

sites having random effects (lines were tested in ~20 locations each year). The percentages of yield benefit across the years appear to be larger compared to previously reported, likely due to different environmental factors such as watering and disease pressures. The yield benefits appear to be stable across years, under different breeding stages and across different performance tests in regional, national (U.S.A), and global scales. The release of RQA for $2N^{v}S$ carriers with both spring and winter growth habits, from multiple breeding programs and continents, provides new resources that can be used to characterize this introgression. Additionally, our data suggests that this translocation is increasing in frequency and is having an impact on wheat productivity.

SUPPLEMENTARY NOTE 6

CYTOLOGICAL KARYOTYPING

Mitotic metaphase chromosomes were prepared by the conventional acetocarmine-squash and non-denaturing fluorescence *in situ* hybridization (ND-FISH) of three repetitive sequence probes was performed using three probes: Oligo-pSc119.2-1 (Tamra-5'-CCGTT TTGTG GACTA TTACT CACCG CTTTG GGGTC CCATA GCTAT-3'), Oligo-pTa535 (AlexaFluor488-5'-GACGA GAACT CATCT GTTAC ATGGG CACTT CAATG TTTTT TAAAC TTATT TGAAC TCCA-3'), and Oligo-pTa713 (AlexaFluor647-5'-AGACG AGCAC GTGAC ACCAT TCCCA CCCTG TCTTA GCGTA ACGCG AGTCG-3'). Polymorphisms detected with respect to the karyotype of Chinese Spring were summarized in **Supplementary Table 24**. Overall, 44 polymorphisms were detected in hexaploid wheat. A hierarchal clustering of the accessions based on the detected polymorphisms indicated that the accessions were largely divided into clusters (**Extended Data Fig. 7c**) that were in agreement with our other phylogenetic analyses (**Fig. 1a,b; Extended Data Fig. 3d**) The most striking karyotypic difference to Chinese Spring was the translocation between chromosomes 5B and 7B (**Fig. 2e-g**). The presence of polymorphic FISH signals on chromosome 5BS between SY Mattis and Arina*LrFor* suggested that the translocated chromosomes may have different origins or diversified after the translocation event (**Supplementary Table 24**).

Some of the structural rearrangements detected by sequence comparisons (**Extended Data Fig. 1**) and Hi-C (**Supplementary Table 23**) are supported by the karyotyping (**Supplementary Table 24**) and *LTR_Angela* analysis (**Supplementary Tables 13-16**). Namely, a large inversion detected on chromosomes 4B was represented by absence of a block of pTa713 signal on the short arm in Arina*LrFor*, CDC Landmark, CDC Stanley, LongReach Lancer, Robigus, and Paragon. Those without inversions were Julius, Norin 61, Jagger, SY Mattis, Mace, Cadenza, Claire, and Weebill 1. An inversion in the distal region of 3DL in CDC Landmark and CDC Stanley made the distance between two pTa535 signals larger than in other accessions. Inversions at the distal end of 4AL may not be a simple inversion, but associated with loss of FISH signals (loss of pSc119 signal in Norin 61, Julius, Claire, and SY Mattis; loss of pTa713 signal in CDC Stanley). Only Arina*LrFor* was lacking a pTa535 signal at the distal end of chromosome 1AS, which we detected as variable TEs and is consistent with local alignment and *RLC_Angela* analyses (**Supplementary Table 14**). The pTa535 signal at the distal region of 3DL in LongReach Lancer shifted proximal, likely due to insertion of *Th. ponticum* chromatin (**Fig. 2a**). Together, these cytological observations provide experimental evidence to support the observed differences we observed between the RQA.

SUPPLEMENTARY NOTE 7

INHERITANCE OF THE 5B/7B TRANSLOCATION IN NORTH-WESTERN EUROPEAN WHEAT VARIETIES

A panel of 538 north-west European wheat varieties was genotyped for the presence of the chromosome 5B/7B translocation using representative marker *RAC875_c5965_1153* from the Illumina 90K wheat SNP array. These lines include almost the entire diversity of the UK wheat gene pool from the 1920s to the present day, together with their northern European ancestors and relatives. The translocation is widespread, being found in 66% of all lines, ranging from 50% of varieties released before 1970 to 73% of varieties released in the 2000s. Currently, 66% of post-2010 varieties have the translocation. 90% of all lines in the panel that contain the introgression have traceable pedigrees through at least one ancestor. With one or two possible minor exceptions, the ancestry of the translocation in all of these lines can be traced back to French landraces (e.g. “Saumur” types) through early 20th century German (“Heines Kolben”) or French (“Vilmorin-23”, “Vilmorin-27”) ancestral varieties, strongly suggesting that the source of the translocation in modern European wheat germplasm is north-western European landraces. The translocation is absent in subsequent introgressions from other grass species and parents of other wide crosses. We investigated whether the high prevalence of the translocation in this European material might be a result of indirect selection by wheat breeders, using a subset of 135 varieties whose parents are known, were genotyped with the 90K wheat SNP array, and differ for the presence of the translocation. 74/135 of these varieties (55%) inherited the translocation, not significantly different than by chance (p=0.15). Breaking this down by decade of release, the most extreme decade is the 2000s, where 22/35 varieties with different parents have the translocation (p=0.09). However, we have previously shown across the whole pedigree that for varieties with simple biparental parentage, breeders have strongly selected for the favourable parent. To account for this possible co-factor, the translocation analysis was recalculated, taking into account the overall bias towards one parent in each cross. Again, no significant effect was detected, whether across the whole pedigree (p=0.10, 1,000 simulations), or in any particular decade (2000s, p=0.16, 1,000 simulations). It is also possible that the presence of the translocation itself may be deleterious and be simultaneously selected for by breeders due to linkage to advantageous haplotypes. Results from an 8-parent UK multiparent advanced generation intercross (MAGIC) population that was genotyped with the same 90K wheat SNP array, which has not been subjected to breeder selection, suggest that the translocation is perfectly neutral (5/8 =62.5% of parents have the translocation, 62.1% of progeny have it). Furthermore, in genome wide association scan (GWAS) studies using the above wheat panel, we have never detected a QTL associated with the haplotype containing *RAC875_c5965_1153*. In summary, the 5B/7B major translocation of almost a whole chromosome arm appears to be selectively neutral, both naturally and with respect to selection by breeders, and likely owes its high frequency in north-western European germplasm to its presence in the landraces commonly used in the earliest European plant breeding programs.

SUPPLEMENTARY NOTE 8

HAPLOTYPE BLOCK ANALYSES AND VISUALIZATION

Genotyping and marker assisted selection have quickly become standard practice in most modern wheat breeding programs. Genotyping involves the use of DNA markers that are in linkage with the gene or locus of interests; however there may be several other genes that are also in linkage with the marker that will also get inherited, many of which will impact crop performance. For breeding purposes, it is important to identify regions of the genome that are similar between lines as well as regions that may be in genetic linkage and are therefore inherited together. Towards this, we compared the genomes of The 10+ Wheat Genomes lines to identify haplotypes that can be used in applied breeding programs.

Haplotype blocks were calculated using a combination of whole chromosome level pairwise alignments using MUMmer v4.0 and gene-based pairwise alignments using BLAST v2.8. For the MUMmer alignments, chromosome level pairwise alignments between all RQA were performed for each chromosome. For alignments between RQA and scaffold-level assemblies, scaffolds were filtered to contain at least one RefSeqv1.0 gene model projection from the corresponding chromosome. Pairwise alignments between scaffold-level assemblies were performed to the RQA, but not to each other. Haplotype blocks from the MUMmer alignments were called using the script assign_mummer_blocks_whole_genome.r. All scripts used for haplotype construction were deposited to github (https://github.com/Uauy-Lab/pangenome-haplotypes). For the zoomed in haplotypes surrounding the *Sm1* gene (**Fig. 3b**), blocks were defined as above but using a reduced bin size of 250 kb. To complement the MUMmer alignments and allow for direct pairwise comparisons between scaffold-level assemblies, pairwise BLAST alignments of projected genes +/- 2000 bp were conducted for all genome assemblies. Alignments were filtered to remove any alignments containing Ns in the aligned sequence. For each pairwise comparison, gene-based alignments were ordered based on the Chinese Spring RefSeqv1.0 physical position. Haplotype blocks were then called using a sliding window approach using the script assign_BLAST_blocks_whole_genome.r. Haplotype blocks called using MUMmer v4.0 and BLAST were combined using the script combine_mummer_and_BLAST.r. To account for slight differences in the absolute positions of haplotype blocks in **Fig. 3b**, chromosomes were scaled according to the largest chromosome 2B across the RQA, and the coordinates of the haplotype blocks were averaged across the assemblies. The positions of the *Sm1* gene and associated markers was determined using BLAST alignments of the gDNA and marker sequence, respectively, against all assemblies. A database and interactive visualization of the haplotypes has been made available to facilitate gene discover and breeding efforts (http://www.crop-haplotypes.com/).

To complement the haplotype database, we constructed additional genome visualization tools for examining larger structural variation. Pairwise gene comparisons by BLASTn were combined into larger blocks using MCScanX v2.0 and the annotated positions of the projected gene annotations. The data was then imported into AccuSyn (https://accusyn.usask.ca/) and SynVisio (https://synvisio.github.io/#/) visualization tools, with menu options to select genomes for pairwise comparison (https://kiranbandi.github.io/10wheatgenomes/). Pretzel (https://github.com/plantinformatics/pretzel) was also used to visualize and compare the RQA and the projected gene annotations (http://10wheatgenomes.plantinformatics.io/). These tools provide access to linear, multi-dimensional, and circular visualizations comparing the RQA, as well as options to upload additional data tracks by research scientists and breeders using these genomes.
